# A *Trem2*^R47H^ mouse model without cryptic splicing drives age- and disease-dependent tissue damage and synaptic loss in response to plaques

**DOI:** 10.1186/s13024-023-00598-4

**Published:** 2023-02-17

**Authors:** Kristine M. Tran, Shimako Kawauchi, Enikö A. Kramár, Narges Rezaie, Heidi Yahan Liang, Jasmine S. Sakr, Angela Gomez-Arboledas, Miguel A. Arreola, Celia da Cunha, Jimmy Phan, Shuling Wang, Sherilyn Collins, Amber Walker, Kai-Xuan Shi, Jonathan Neumann, Ghassan Filimban, Zechuan Shi, Giedre Milinkeviciute, Dominic I. Javonillo, Katelynn Tran, Magdalena Gantuz, Stefania Forner, Vivek Swarup, Andrea J. Tenner, Frank M. LaFerla, Marcelo A. Wood, Ali Mortazavi, Grant R. MacGregor, Kim N. Green

**Affiliations:** 1 Department of Neurobiology and Behavior, University of California, Irvine, USA; 2 Institute for Memory Impairments and Neurological Disorders, University of California, Irvine, USA; 3 Transgenic Mouse Facility, Office of Research, ULAR, Irvine, USA; 4 Department of Developmental and Cell Biology, University of California, Irvine, USA; 5 Center for Complex Biological Systems, Irvine, USA; 6 Department of Pharmaceutical Sciences, University of California, Irvine, USA; 7 Department of Molecular Biology & Biochemistry, University of California, Irvine, USA; 8 Department of Pathology and Laboratory Medicine, University of California, Irvine, USA

**Keywords:** Alzheimer’s Disease, Microglia, Inflammation, TREM2 R47H, MODEL-AD, LTP

## Abstract

**Background:**

The TREM2 R47H variant is one of the strongest genetic risk factors for late-onset Alzheimer’s Disease (AD). Unfortunately, many current *Trem2* ^R47H^ mouse models are associated with cryptic mRNA splicing of the mutant allele that produces a confounding reduction in protein product. To overcome this issue, we developed the *Trem2*^R47H NSS^ (Normal Splice Site) mouse model in which the *Trem2* allele is expressed at a similar level to the wild-type *Trem2* allele without evidence of cryptic splicing products.

**Methods:**

*Trem2*^R47H NSS^ mice were treated with the demyelinating agent cuprizone, or crossed with the 5xFAD mouse model of amyloidosis, to explore the impact of the TREM2 R47H variant on inflammatory responses to demyelination, plaque development, and the brain’s response to plaques.

**Results:**

*Trem2*^R47H NSS^ mice display an appropriate inflammatory response to cuprizone challenge, and do not recapitulate the null allele in terms of impeded inflammatory responses to demyelination. Utilizing the 5xFAD mouse model, we report age- and disease-dependent changes in *Trem2*^R47H NSS^ mice in response to development of AD-like pathology. At an early (4-month-old) disease stage, hemizygous 5xFAD/homozygous *Trem2*^R47H NSS^ (5xFAD/*Trem2*^R47H NSS^) mice have reduced size and number of microglia that display impaired interaction with plaques compared to microglia in age-matched 5xFAD hemizygous controls. This is associated with a suppressed inflammatory response but increased dystrophic neurites and axonal damage as measured by plasma neurofilament light chain (NfL) level. Homozygosity for *Trem2*^R47H NSS^ suppressed LTP deficits and loss of presynaptic puncta caused by the 5xFAD transgene array in 4-month-old mice. At a more advanced (12-month-old) disease stage 5xFAD/*Trem2*^R47H NSS^ mice no longer display impaired plaque-microglia interaction or suppressed inflammatory gene expression, although NfL levels remain elevated, and a unique interferon-related gene expression signature is seen. Twelve-month old *Trem2*^R47H NSS^ mice also display LTP deficits and postsynaptic loss.

**Conclusions:**

The *Trem2*^R47H NSS^ mouse is a valuable model that can be used to investigate age-dependent effects of the AD-risk R47H mutation on TREM2 and microglial function including its effects on plaque development, microglial-plaque interaction, production of a unique interferon signature and associated tissue damage.

**Supplementary Information:**

The online version contains supplementary material available at 10.1186/s13024-023-00598-4.

## Background

Triggering receptor expressed on myeloid cells 2 (TREM2) is an immunomodulatory cell surface receptor expressed in myeloid lineage cells including microglia [1, 2]. TREM2 has been implicated in functioning in a wide range of processes including cell proliferation, survival, phagocytosis and regulation of inflammation [3, 4]. TREM2 can bind many polyanionic ligands including bacterial lipopolysaccharide, lipoproteins and phospholipids whose presence are often associated with infection or cellular damage [5]. TREM2 is expressed as a single pass transmembrane protein with an extracellular V-type immunoglobulin domain and a short ectodomain (aa 1–174), a single transmembrane helix and a short cytoplasmic sequence (aa 196–230). TREM2 can also be produced as a soluble extracellular protein via alternative splicing or via proteolytic processing of the transmembrane form within the ectodomain. Intracellular signaling via TREM2 receptor can be mediated via DNAX-activating protein of 12 kDa (DAP12, also known as TYROBP) as well as DAP-10 which can propagate downstream intracellular signaling via spleen tyrosine kinase (SYK) and phosphatidylinositol 3-kinase (PI3K). Mechanisms of signaling via TREM2 in vivo have not yet been fully elucidated.

Mutations within *TREM2* can be associated with age-dependent development of several neurodegenerative diseases depending on the specific variant [6–10]. A complete loss of function of TREM2 or DAP-12 results in Nasu-Hakola disease, a rare inherited leukodystrophy characterized by bone cysts, bone fractures and sclerosing leukoencephalopathy associated with progressive pre-senile dementia [11, 12]. More recently, human genome-wide association studies (GWAS) identified the relatively rare R47H missense variant in *TREM2* as being strongly and reproducibly linked to increased risk of development of Late-Onset AD (LOAD; [13, 14]). The R47 residue is located within a poly-basic region of the extracellular Ig-like domain, and likely modifies interactions of TREM2 with its ligands such as phospholipids, apolipoproteins, apoptotic neurons and Aβ [15, 16].

*TREM2* is primarily expressed by microglia, the primary immune cells of the central nervous system that play important roles in responding to pathological insults and maintaining tissue homeostasis. Moreover, these cells have been strongly implicated in the development of LOAD. During AD, microglia mount an inflammatory response to Aβ plaques as evidenced by findings in both human AD brains and animal models of the disease [17, 18]. Accumulating evidence implicates microglia in several AD-related processes including plaque formation and growth [19], plaque compaction [19, 20], constituting a protective barrier against dystrophic neurites [21], promoting or preventing development and spreading of Tau pathology [22], cerebral amyloid angiopathy [19], destruction of perineuronal nets [23, 24], as well as synaptic and neuronal loss [23, 25–31]. Understanding how alteration in TREM2^R47H^ function contributes to AD should provide important insight into how microglial biology contributes to AD.

The mouse is a powerful model mammalian genetic system to investigate consequences of human genetic dysfunction on development and homeostasis, including AD [32, 33]. Three groups used CRISPR/Cas9 to develop mouse models to investigate the properties of the R47H mutation on TREM2 function in vivo [34–36]. Unfortunately, in all three cases, generation of *Trem2*^R47H^ mouse models via CRISPR produced unintended cryptic splicing products from the mutant *Trem2* allele, resulting in dramatically reduced level of TREM2 protein not observed in human R47H carriers, which confounded alignment of phenotypes observed in mice with those in humans [34, 35]. We have overcome this limitation by developing a *Trem2*^R47H^ mouse model without cryptic splicing and with normal level of transcription of the *Trem2*^R47H^ allele. We characterized the response of mice homozygous for this new *Trem2*^R47H^ allele to acute demyelination following administration of cuprizone and compared this with the response observed in mice homozygous for a cryptic splice site allele of *Trem2*^R47H^ as well as mice with a null allele of *Trem2*. We also investigated the consequence of homozygosity for the new *Trem2*^R47H^ allele on development of pathology using the 5xFAD mouse model at early and late stages of disease progression. This new mouse *Trem2*^R47H^ model can be used to investigate mechanisms whereby the TREM2 R47H mutation contributes to development of human late onset AD.

## Methods

### Animals

All experiments involving mice were approved by the UC Irvine Institutional Animal Care and Use Committee and were conducted in compliance with all relevant ethical regulations for animal testing and research. All experiments involving mice comply with the Animal Research: Reporting of in Vivo Experiments (ARRIVE) guidelines.

### Mice

To generate *Trem2*^R47H^ mice (B6(SJL)-*Trem2*^*em1Aduci*^/J, Jackson Laboratory stock number #034,036), Alt-R Crispr RNA (TMF1342 – gaagcactgggggagacgca) and tracrRNA plus CAS9 protein (HiFi Cas9 nuclease V3, Integrated DNA Technologies (IDT), Coralville, IA) as a ribonucleoprotein (RNP) was microinjected into C57BL/6 J zygotes (Jackson Lab Stock # 000,664) along with a ssODN sequence (TMF 1341–5’-CAAGCCCTCAACACCACGGTGCTGCAGGGCATGGCCGGCCAGTCGTTAAGGGTATCCTGCACTTATGACGCGTTGAAACATTGGGGCAGACATAAGGCCTGGTGTCGGCAGCTGGGTGAGGAGGGCCCATGCCAGCGTGTGGT—3’) to introduce the R47H missense mutation. G0 founder animals containing the desired DNA sequence changes were backcrossed with C57BL/6 J mice and N1 heterozygous mice were sequenced to determine the mutant allele. N1 heterozygous mice were backcrossed to produce N3F1 heterozygotes, which were used to generate animals for subsequent analysis. 5xFAD hemizygous (B6.CgTg(APPSwFlLon,PSEN1*M146L*L286V)6799Vas/Mmjax, Jackson Lab Stock # 34,848, MMRRC) and non-transgenic littermates were produced by natural mating or IVF procedures with C57BL/6 J females. After weaning, they were housed together with littermates and aged until the harvest dates. All animals were bred by the Transgenic Mouse Facility at UCI.

### Off-target analysis

Genomic DNA was extracted from mouse tail biopsies using DirectPCR Lysis Reagent (Viagen Biotech, Los Angeles, CA) and Proteinase K (Roche, Indianapolis, IN). Amplification was performed using a Bio-Rad CFX-96 instrument. For each amplicon, a single PCR product was confirmed by capillary electrophoresis (Fragment Analyzer, AATI / Agilent, Santa Clara, CA) then subjected to Sanger sequencing (Retrogen, San Diego, CA) and analyzed using SeqMan Pro 17.2 (DNASTAR, Madison, WI). Primers for PCR amplification and sequencing for off-target analysis are listed in Supplemental Table 1).

### Genotyping

Oligonucleotides for PCR-based genotyping were purchased from IDT. *Trem2*^R47H^ ^NSS^ genotyping was performed using a common primer set to amplify both *Trem2* wildtype allele and *Trem2*^R47H^ ^NSS^ allele (For 5′-TCAACACCACGGTGCT -3′ and Rev 5′-TGTGTGCTCACCACACG -3′). Two fluorophore labeled-hydrolysis probes which hybridized specific to mouse *Trem2* wildtype amplicon (5’-TGCGTCTCCCCCAGTGCTTCAA-3’ + HEX) and *Trem2*^R47H NSS^ mutation (5’-TATGTCTGCCCCAATGTTTCAACGCG-3’-FAM) were used to detect the allelic ratio in the amplicon. The relative fluorescence from each probe was quantified at the end point of PCR cycles to call the genotype using Allelic Discrimination function using Bio-Rad CFX Maestro software (Bio-Rad, Hercules, CA). For 5xFAD genotyping, a hydrolysis probe which hybridizes to APP(Swe) mutation amplicon was used (For 5′-TGGGTTCAAACAAAGGTGCAA-3′ and Rev 5′-GATGACGATCACTGTCGCTATGAC-3′: APP(Swe) probe 5′-CATTGGACTCATGGTGGGCGGTG-3′.) to detect transgenes. We used endogenous *ApoB* allele (For 5′-CACGTGGGCTCCAGCATT-3′ and Rev 5′-TCACCAGTCATTTCTGCCTTTG-3′: *ApoB* probe 5′-CCAATGGTCGGGCACTGCTCAA-3′) to normalize the Ct values.

### Cuprizone (CPZ) treatment

Eight-week-old C57BL/6 J, *Trem2*^em1Aduci^ (i.e. *Trem2*^R47H NSS^), *Trem2*^em1Adiuj^ (i.e. *Trem2*^R47H CSS^), and *Trem2*^em2Adiuj^ (i.e. *Trem2*-KO) mice (Jackson Laboratory stock number: 000664, 034,036, 027,918, 027,197 respectively) were used. Each mouse model consisted of 2 groups of 4 male mice. Control mice were supplied standard chow for 6 weeks while the CPZ groups were fed 0.3% cuprizone chow also for 6 weeks (Envigo, Indianapolis, IN). Mice within the same experiment group (i.e., same genotype and diet) were housed together for the duration of feeding. Weights of individual mouse and chow consumptions of each cage were recorded, and chow was changed every 3 or 4 days to monitor expected weight loss as well as ensuring freshness of cuprizone chow. Brains were collected and fixed in 4% paraformaldehyde for 24 h followed by cryoprotection by immersion in 5% sucrose for 24 h then 30% sucrose for 5 days, all at 4 °C.

### Histology

Mice were euthanized at 4 and 12 months of age via CO_2_ inhalation and transcardially perfused with 1X phosphate buffered saline (PBS). For all studies, brains were removed, and hemispheres separated along the midline. Brain halves were either flash frozen for subsequent biochemical analysis or drop-fixed in 4% paraformaldehyde (PFA (Thermo Fisher Scientific, Waltham, MA)) for immunohistochemical analysis. Fixed half brains were sliced at 40 μm using a Leica SM2000R freezing microtome. All brain hemispheres were processed and coronal brain slices (between -2.78 mm posterior and –3.38 mm posterior to Bregma according to the Allen Mouse Brain Atlas, Reference Atlas version 1, 2008) imaged with a Zeiss Axio Scan Z1 Slidescanner using a 10X 0.45 NA Plan-Apo objective. Images were also acquired using a 20X objective via a Leica TCS SPE-II confocal microscope and quantified using Bitplane Imaris Software.

### Immunohistochemistry

One representative brain slice from each mouse of the same experimental group (i.e. same genotype, age, and sex) was stained in the same well. Free-floating sections were washed three times with 1X PBS (1 × 10 min and 2 × 5 min) and for Thioflavin-S (Thio-S) staining, 10 min incubation in 0.5% Thio-S (1892; Sigma-Aldrich) diluted in 50% ethanol. Sections were then washed 2X for 5 min each in 50% ethanol and one 10-min wash in 1xPBS. For Amylo-Glo staining, following the PBS washes, free-floating brain slices were washed in 70% ethanol for 5 min and rinsed in deionized water for 2 min before being immersed for 10 min in Amylo-Glo RTD Amyloid Plaque Staining Reagent (1:100; TR-200-AG; Biosensis, Thebarton, South Australia) diluted in 0.9% saline solution. Afterwards, sections were washed in 0.9% saline solution for 5 min then rinsed in deionized water for 15 s before proceeding with a standard indirect immunohistochemical protocol. From the incubation period for both Thio-S and Amylo-Glo onwards, sections were kept under foil or in the dark. Sections were immersed in normal blocking serum solution (5% normal goat serum with 0.2% Triton X-100 in 1X PBS) for 1 h before overnight incubation at 4 °C in primary antibodies diluted in normal blocking serum solution.

Brain sections were stained following a standard indirect technique as described [37, 38] with the following primary antibodies against: ionized calcium-binding adapter molecule 1 (IBA1; 1:2000; 019–19,741; Wako, Osaka, Japan), Aβ_1-16_ (6E10; 1:2000; 8,030,001; BioLegend, San Diego, CA), glial fibrillary acidic protein (GFAP; 1:1000; AB134436; Abcam, Cambridge, MA), S100β (1:200; AB41548; Abcam), lysosome-associated membrane protein 1 (LAMP1; 1:200; AB25245, Abcam), neurofilament light chain (NfL; 1:200; 171 002; Synaptic Systems, Germany), CD74 (1:500; 151,002; BioLegend), CD11c (1:100.; 50–112-2633; eBioscience), myelin basic protein (MBP; 1:200; MAB386; (Millipore Sigma, Billerica, MA), Synaptophysin (1:1000; S5768; Sigma-Aldrich), PSD-95 (1:500; ab18258; Abcam), Bassoon (BSN; 1:250; AB82958; Abcam), OC (1:1000; AB2286; Sigma-Aldrich). Brain sections were mounted on microscope slides, dried overnight and desiccated for one hour prior to standard Luxol Fast Blue (LFB) staining protocols [39].

High-resolution fluorescence images were obtained using a Leica TCS SPE-II confocal microscope and LAS-X software. For confocal imaging, one field of view (FOV) per brain region was captured per mouse using the Allen Brain Atlas to capture comparable brain regions.

For super-resolution imaging, images of the CA1 stratum radiatum (CA1-SR) hippocampal region of WT, *Trem2*^R47H NSS^, 5xFAD and 5xFAD/*Trem2*^R47H NSS^ animals at 4 and 12 months of age were acquired with identical conditions. Super-Resolution Lattice Structured Illumination Microscopy (Lattice-SIM) was performed using an Elyra 7 microscope system (Carl Zeiss, White Plains, NY). Samples were imaged using a 63 × 1.4NA Plan-Apo objective lens and Immersol 518F (23°C) immersion oil. Images were collected as z-stacks (110 nm step interval, within a depth of 5–8 µm, covering an area of 64 × 64 µm) and for each focal plane, 9 phase images were acquired. Images were then processed using ZEN SIM^2^ (weak fixed option selected) on the ZEN (black edition) software. Two images per mouse/genotype/age/sex were acquired.

### Quantification of soluble and insoluble fraction Aβ and neurofilament light chain

Preparation of samples and quantification of Aβ was performed as described [37, 38]. Micro-dissected hippocampal and cortical regions of each mouse were flash-frozen and processed for biochemical analysis. Samples were pulverized using a Bessman Tissue Pulverizer. Pulverized hippocampal tissue separated for biochemical analysis was homogenized in 150µL of Tissue Protein Extraction Reagent (TPER; Life Technologies, Grand Island, NY), while cortical tissue was homogenized in 1000µL/150 mg of TPER. This composition of TPER includes 25 mM bicine and 150 mM sodium chloride (pH 7.6) to efficiently solubilize proteins within brain tissue following homogenization. Together with protease (Roche) and phosphatase inhibitors (Sigma-Aldrich), the homogenized samples were centrifuged at 100,000 g for 1 h at 4 °C to generate TPER-soluble fractions. For formic acid-fractions, pellets from TPER-soluble fractions were homogenized in 70% formic acid: 75µL for hippocampal tissue or half of used TPER volume for cortical tissue. Afterwards, samples were centrifuged again at 100,000 g for 1 h at 4 °C. Protein in the insoluble fraction of micro-dissected hippocampal and cortical tissue were normalized to its respective brain region weight, while protein in soluble fractions were normalized to the protein concentration determined via Bradford Protein Assay. Formic acid neutralization buffer was used to adjust pH prior to running ELISAs.

Quantitative biochemical analyses of human Aβ soluble and insoluble fraction levels were acquired using the V-PLEX Aβ Peptide Panel 1 (6E10) (K15200G-1; Meso Scale Discovery, Rockville, MD). Quantitative biochemical analysis of neurofilament-light chain (NfL) in plasma was performed using the R-Plex Human Neurofilament L Assay (K1517XR-2; Meso Scale Discovery).

### Imaris quantitative analysis

Confocal images of each brain region were quantified automatically using the spots module within Imaris v9.7 then normalized to the area of the field-of-view (FOV). Amyloid burden was assessed by measuring both the total Thio-S^+^ plaque number normalized to FOV area and their volume via the surfaces module in Imaris. Similarly, volumetric measurements (i.e., Thio-S^+^ plaque volume, IBA1^+^ microglia volume, etc.) were acquired automatically utilizing the surfaces module with confocal images of each brain region. Quantitative comparisons between experimental groups were carried out in sections stained simultaneously.

For synaptic quantification, the total number of Bassoon or PSD95 puncta was quantified using the spots function on Imaris. Co-localization of pre- and post-synaptic puncta (Bassoon-PSD95) was determined using the spots function on Imaris and Matlab software to determine the total number of colocalized spots (defined as ≤ 200 nm distance). Results were normalized to the total volume of each image, to correct for any difference in the depth of imaging.

### Long-term potentiation

Hippocampal slices were prepared from WT, homozygous *Trem2*^R47H NSS^, hemizygous 5xFAD, 5xFAD/ *Trem2*^R47H NSS^ mice (1–2 slice recordings/mouse for 5–6 mice/sex/genotype totaling to *n* = 8–12 slices/sex/genotype) at 4 and 12 months of age. Hippocampal slice preparation and long-term potentiation (LTP) recording was performed as described [37, 38]. Following isoflurane anesthesia, mice were decapitated, and the brain was quickly removed and submerged in ice-cold, oxygenated dissection medium containing (in mM): 124 NaCl, 3 KCl, 1.25 KH_2_PO_4_, 5 MgSO_4_, 0 CaCl_2_, 26 NaHCO_3_, and 10 glucose. Coronal hippocampal slices (340 µm) were prepared using a Leica VT1000S vibrating tissue slicer before being transferred to an Interface recording containing preheated artificial cerebrospinal fluid (aCSF) of the following composition (in mM): 124 NaCl, 3 KCl, 1.25 KH_2_PO_4_, 1.5 MgSO4, 2.5 CaCl_2_, 26 NaHCO_3_, and 10 glucose and maintained at 31 ± 1 °C. Slices were continuously perfused with this solution at a rate of 1.75–2.00 ml/min while the surface of the slices were exposed to warm, humidified 95% O_2_ / 5% CO_2_. Recordings began following at least 2 h of incubation. Field excitatory postsynaptic potentials (fEPSPs) were recorded from CA1b striatum radiatum using a single glass pipette filled with 2 M NaCl (2–3 MΩ) in response to orthodromic stimulation (twisted nichrome wire, 65 µm diameter) of Schafer collateral-commissural projections in CA1 striatum radiatum. Pulses were administered at 0.05 Hz using a current that elicited a 50% maximal response. Paired-pulse facilitation was measured at 40, 100, and 200 s intervals prior to setting baseline. After establishing a 20-min stable baseline, the orthodromic stimulated pathway was used to induce LTP by delivering 5 ‘theta’ bursts, with each burst consisting of four pulses at 100 Hz and the bursts themselves separated by 200 ms (i.e., theta burst stimulation (TBS)). The stimulation intensity was not increased during TBS. Data were collected and digitized by NAC 2.0 Neurodata Acquisition System (Theta Burst Corp., Irvine, CA).

### RNA sequencing

RNA sequencing was performed as described [37]. Frozen tissues were lysed and homogenized using TissueLyser II (Qiagen, Germantown, MD). Total RNAs were extracted using RNeasy Mini Kit (Qiagen) and RNase-Free DNase Set (Qiagen) on a QIAcube (Qiagen) liquid handling platform. RNA integrity number (RIN) was measured by Qubit RNA IQ Assay (Life Technologies) and samples with RIN >  = 7.0 were kept for cDNA synthesis. cDNA synthesis and amplification were performed followed by Smart-seq2 [40] standard protocol.

Libraries were constructed using the DNA Prep Kit (Illumina, San Diego, CA) on an epMotion 5070 TMX (Eppendorf, Enfield, CT) automated pipetting system. The 4 months libraries were constructed manually and the 12 months libraries by the epMotion 5070 TMX (Eppendorf) automated pipetting system using the DNA Prep Kit (Illumina).

Libraries were base-pair selected based on Agilent 2100 Bioanalyzer profiles and normalized determined by KAPA Library Quantification Kit (Roche). The libraries were built from 3 to 5 different mice per genotype, sex and tissue (hippocampus) across 2 different timepoints (4 and 12 months). For the cuprizone experiment, whole brains were used for 4 males per genotype per condition (control or CPZ) at 3 months of age. The 4-month-old mouse libraries were sequenced using paired-end 43 bp mode on Illumina NextSeq500 platform with > 14 million reads per sample. The 12-month-old mouse libraries were sequenced using paired-end 101 bp mode on Illumina NextSeq2000 platform with > 28 million reads per sample. Sequences were aligned to the mouse genome (mm10) and annotation was done using GENCODE v21. Reads were mapped with STAR v.2.7.3a and RSEM (v.1.3.3) was used for quantification of gene expression.

#### Principle component analysis

Principal component analysis of the datasets was calculated in R version 4.5 using the prcomp function of the stats package.

#### Differential gene expression analysis

Differential gene expression analysis was performed using edgeR per timepoint and genotype. Genes with a log2(Fold Change) > 1 and various threshold for value depending on comparisons were labelled. To compare different sets of genes differentially expressed we created a binary matrix identifying up and downregulated genes across different comparisons. A matrix indicating up or downregulation was later used to plot a heatmap. From the comparisons, lists of genes of interest were chosen to plot a heatmap of their expression and a GO term enrichment analysis using enrichR (https://amp.pharm.mssm.edu/Enrichr/) and the top GO terms plotted.

#### Weighted correlation gene network analysis

Weighted gene correlation network analysis (WGCNA) was done using PyWGCNA [41] on two different datasets: 1. *Trem2*^R47H^ dataset with matching controls at 2 different timepoints (4 month and 12 month) in hippocampus for both sexes, 2. Cuprizone and control groups in male brain samples. For both datasets, we used a quantile normalized matrix filtered by genes with more than 1 TPM and without an outlier sample. Based on our datasets, we used power 7 as a soft threshold for first dataset and power 8 for second one. The other parameters were the same for both including: min. module size = 50 and MEDissThres = 0.2. We identified significant modules by calculating the correlation with the traits, then proceeded to plot the behavior per sample of the genes in the white, lightcoral module (first dataset) and darkgrey module (second dataset), by doing a GO term analysis using PyWGCNA.

### Nanopore Sequencing

Nanopore libraries were built from three mice per genotype for C57BL/6 J, *Trem2* ^R47H CSS^ homozygotes and *Trem2* ^R47H NSS^ homozygotes. Each library was constructed from 200 fmol Smartseq2 cDNA using Ligation Sequencing Kit (SQK-LSK114, Oxford Nanopore Technology, (ONT) Lexington, MA) and NEBNext® Companion Module for ONT Ligation Sequencing (E7180S). The Short Fragment Buffer (SFB) from the Ligation kit was used during the wash steps. Each 10 fmol library was loaded on one R10.4.1 flow cell (FLO-MIN114, ONT) and sequenced using a GridIONx5 Mk1B (ONT) with live base calling using Guppy (v6.2.7) run in super accurate mode.

#### Long-read nanopore analysis

Adapters were trimmed from reads with Porechop (v0.2.4) with added custom adapter sequence reflecting the new TSO/nanopore chimeric adapters in the libraries. Reads were aligned to the mouse genome (mm10) minimap2 (v2.24) and the GENCODE v21 annotation. Reads were then sorted with samtools (v1.15.1) and flipped with a custom script. Reference-based error correction was done using TranscriptClean [42] with the following options: –canonOnly –primaryOnly –correctMismatches False. TALON [43] was used to quantify and categorize isoforms using default parameters with the addition of –minCount = 3 for the talon_filter_transcripts module.

### Statistics

Every reported *n* represents the number of independent biological replicates. The sample sizes are similar with those found in prior studies conducted by MODEL-AD and were not predetermined using statistical methods [37, 38]. Electrophysiology, immunohistochemical, and biochemical data were analyzed using Student’s t-test or two-way ANOVA via Prism v.9 (GraphPad, La Jolla, CA). Tukey’s post-hoc tests were utilized to examine biologically relevant interactions from the two-way ANOVA. Where sex-differences are apparent, a Student’s t-test was used within genotype group. Outlier tests were performed via Prism v.9 where relevant and any datapoints removed from the analyses acknowledged in the relevant figure legend. *p ≤ 0.05, ** *p* ≤ 0.01, ***p ≤ 0.001, ****p ≤ 0.0001. Statistical trends were accepted at *p* < 0.10 (^#^). Data are presented as raw means and standard error of the mean (SEM).

## Results

### ***The Trem2***^R47H NSS^ mutation promotes loss of oligodendrocyte gene expression in response to cuprizone treatment.

**Fig. 1:**
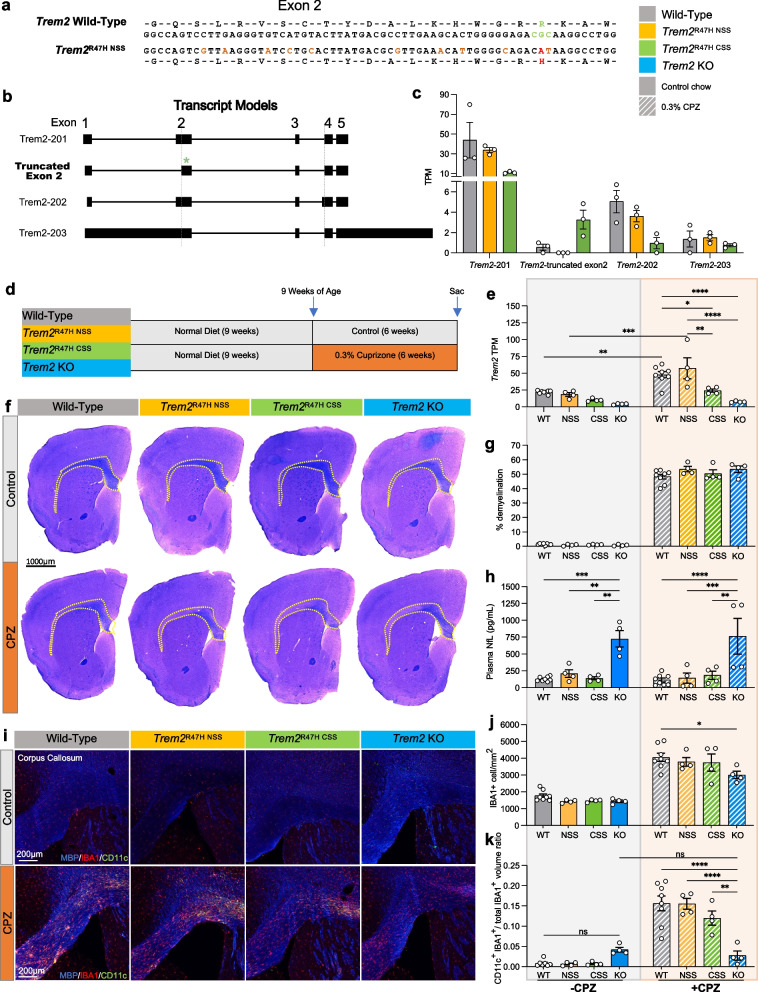
Cuprizone model of demyelination using wild-type, *Trem2*^R47H NSS^, *Trem2*^R47H CSS^, and *Trem2* KO mice. **a** Amino acid coding sequence within exon 2 of mouse wild type *Trem2* and *Trem2* ^R47H NSS^ alleles. The triplet codon for arginine (R, green), the G to A transition that encodes histidine (H, red), and ten silent DNA mutation (tan) are shown in the *Trem2*^R47H NSS^ allele. **b** Transcript models from long-read RNA-seq analysis showing *Trem2* isoforms 201, 202, 203, and the truncated exon 2 from cryptic splicing reported in *Trem2*^R47H CSS^. The green asterisk denotes the truncated exon 2. **c** *Trem2* isoform TPM values of wild-type, *Trem2*^R47H NSS^ and *Trem2*^R47H CSS^. **d** Cuprizone (CPZ) feeding scheme of wild-type, *Trem2*^R47H NSS^, *Trem2*^R47H CSS^ and *Trem2* KO mice. **e** *Trem2* TPM values of wild-type, *Trem2*^R47H NSS^, *Trem2*^R47H CSS^ and *Trem2* KO males examined from whole-brain bulk RNA-seq data showed similar increase in *Trem2* expression in response to CPZ treatment in wild-type and *Trem2*^R47H NSS^ that was attenuated in *Trem2*^R47H CSS^ and *Trem2* KO mice. **f** Representative whole-brain stitched images of wild-type, *Trem2*^R47H NSS^, *Trem2*^R47H CSS^ and *Trem2* KO males on control or CPZ diet stained for myelinated fibers with Luxol Fast Blue. Yellow dotted lines indicate region of demyelination. Scale bar = 1000 µm. **g** Quantification of demyelination reveals no change between genotypes. **h** Plasma neurofilament light-chain (NfL) quantified via Meso Scale Discovery suggests exacerbated axonal damage in *Trem2* KO mice. **i** Representative images of corpus collosum from wild-type, *Trem2*^R47H NSS^, *Trem2*^R47H CSS^ and *Trem2* KO mice on control vs CPZ diet stained for myelin basic protein (blue), microglia (IBA1, red), and DAM gene marker (CD11c, green). **j** Quantification of IBA1^+^ cells per mm^2^ revealed expected increase in microgliosis in response to demyelination in CPZ-treated mice across all groups with *Trem2* KO having fewer microglia than wild-type. **k** Quantification of CD11c^+^ microglia cell volume normalized to microglia volume. n = 4–8. Data are represented as mean ± SEM. Two-way ANOVA followed by Tukey’s post-hoc tests to examine biologically relevant interactions. Statistical significance is denoted by *p < 0.05, **p < 0.01, ***p < 0.001, ****p < 0.0001

Results of previous studies of mice with the *Trem2*^R47H^ missense mutation introduced via CRISPR suggested that it acts as a near-complete loss of function, recapitulating phenotypes seen in *Trem2* knock-out (KO) mice [34, 36]. However, subsequent analyses of *Trem2* expression and splicing in these models identified the unexpected generation of a cryptic splice site and subsequent reduction of *Trem2* expression due to the synonymous codon changes introduced as part of the CRISPR repair template [35]. Given the importance of a *Trem2*^R47H^ mouse model that more accurately recapitulates human cases, we designed alternative CRISPR repair templates, guided in part by Cheng et al., to introduce the R47H mutation into C57BL/6 J mice [44]. Confirmation of the desired sequence change (CGC – > CAT; arginine – > histidine) and synonymous silent codon changes are shown in Fig. 1a. To analyze expression and isoform usage of *Trem2* from the *Trem2*^R47H NSS^ allele, we extracted RNA from whole brains of 15 week old wild-type and *Trem2*^R47H NSS^ mice, as well as from a cohort of *Trem2*^R47H CSS^ mice with the identified Cryptic Splice Site (CSS) and reduced expression [35] and conducted Nanopore long-read RNA-seq to examine the association of exons within individual transcripts. We identified three known annotated *Trem2* isoforms – *Trem2-201* encoding transmembrane TREM2, *Trem2-*202 encoding secreted TREM2, and *Trem2-*203 which contains retained introns (Fig. 1b, Supplemental Fig. 1). However, a novel truncated exon 2 containing isoform was abundant in the *Trem2*^R47H CSS^ brain and was not present in *Trem2*^R47H NSS^ mice. We designated our model as *Trem2*^R47H NSS^ (Normal Splice Site, NSS; available at The Jackson Laboratory—stock #034,036). Furthermore, reads of the major isoform, *Trem2*-201, are reduced in the *Trem2*^R47H CSS^ brains (> 50% reduction—wild-type mean TPM = 43.7, NSS = 33.8, CSS = 11.03; Fig. 1c).

To further validate the *Trem2*^R47H NSS^ model we conducted an off-target analyses of any other putative cut sites that might have been targeted by CRISPR-Cas9 during generation of the *Trem2*^R47H NSS^ allele. *Trem2*^R47H NSS^ founder mice were backcrossed with wild type C57BL/6 J animals for three generations before use to generate animals for this study, making it unlikely that a mutation caused by an off-target effect of CRISPR/Cas9 would be present on a chromosome other than chromosome 17, i.e., the location of *Trem2* (C57BL/6 J; Chr17; 48.6 Mb, GRCm39, Ensembl release 108). Potential CRISPR/Cas9 off-target sites with up to four mismatches using crRNA TMF1342 were screened for using Cas-OFFinder (http://www.rgenome.net/cas-offinder/; [45]). Eleven potential off-target sites on mouse chromosome 17 were identified (Supplemental Table 2). Two potential off-target sites were within coding exons of genes, six were within introns, while the remaining three were within intergenic regions. To screen for evidence of CRISPR/Cas9 RNP activity at each site, DNA from a wild-type and homozygous *Trem2*^R47H NSS^ mouse was amplified by PCR using the primers listed (Supplementary Table 1) then sequenced across the potential off-target region at each locus (Supplemental Fig. 2a-k). None of the 11 potential off-target sites showed a difference in sequence between WT and homozygous *Trem2*^R47H NSS^ mice. Moreover, no significant change in gene expression was seen in any of these 11 genes via RNA-seq from whole brain extracted RNA (Supplemental Table 2). We also explored transcript isoform production from these genes through long-read RNA-seq and found no change (e.g. data for *Zpf945* shown in Supplemental Fig. 2l, m).

Having confirmed correct expression, isoform usage, and an absence of off-target genomic effects, we next assessed the impact of the *Trem2*^R47H NSS^ variant on microglial function and tested if it acts as a loss of function allele using a cuprizone model of demyelination [46]. Both myelin and fibrillar Aβ act as TREM2 ligands and induce a distinctive gene expression profile in microglia known as a Disease Associated Microglia (DAM) signature, which can be explored with cuprizone. For comparison, we included cohorts of *Trem2*^R47H CSS^ mice, as well as *Trem2* KO mice. These 3 groups and a wild-type group, were treated with cuprizone (0.3%) or control chow for 6 weeks (Fig. 1d), then were euthanized at 15 weeks and brains examined by histology and bulk RNA-seq. Only male mice were used for the cuprizone study due to the influence of estrogen and progesterone on myelination, as well as evidence of the treatment disrupting estrous cycle in mice [47, 48]. RNA was extracted from half brains, and bulk RNA-seq conducted, with a principal component analysis (PCA) plot for all samples shown in Supplemental Fig. 3a. *Trem2* expression values were plotted, showing that *Trem2*^R47H NSS^ mice have similar *Trem2* expression levels to wild-type mice consistent with results of the long-read RNA-seq analysis, and that both *Trem2*^R47H CSS^ and *Trem2* KO mice have reduced expression (Fig. 1e). Notably, with cuprizone treatment, *Trem2* levels increased similarly in wild-type and *Trem2*^R47H NSS^ mice, but to a lesser extent in *Trem2*^R47H CSS^ mice.

Luxol Fast Blue staining for myelin confirmed demyelination in all cuprizone treated mice with no overt difference in the extent of demyelination between the four groups (Fig. 1f, g). Plasma neurofilament light-chain (NfL) reflects axonal damage in the brain [49]. Interestingly, cuprizone treatment/demyelination did not increase plasma NfL in either WT mice, or the *Trem2* variants/KO, although a substantial elevation of plasma NfL was present in *Trem2* KO mice independent of cuprizone treatment (Fig. 1h). Immunofluorescence analysis for microglia (IBA1) and DAM marker CD11c (encoded by *Itgax*) reveals extensive microgliosis in the corpus callosum of cuprizone treated wild-type, *Trem2*^R47H NSS^, and *Trem2*^R47H CSS^ mice, and to a lesser extent *Trem2* KO mice (Fig. 1i, j). While CD11c expression is induced in cuprizone treated wild-type, *Trem2*^R47H NSS^, and *Trem2*^R47H CSS^ mice, no induction is observed in the *Trem2* KO mice (Fig. 1k).

**Fig. 2:**
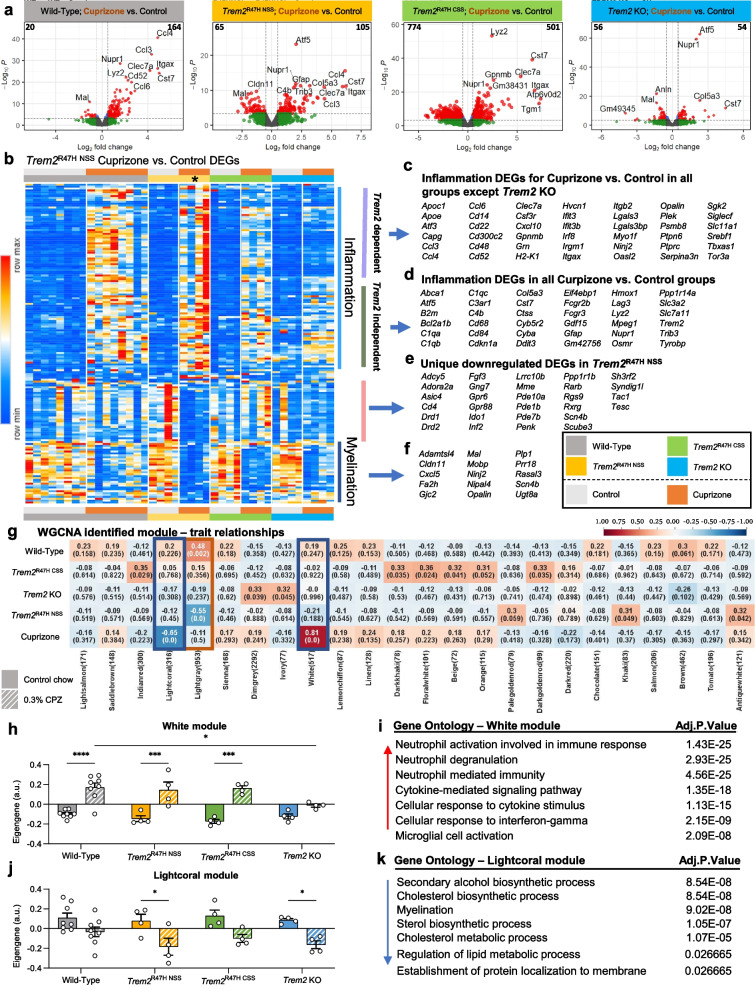
*Trem2*^R47H NSS^ induces increased inflammatory response but reduces myelination gene expression in response to cuprizone. **a** Volcano plot of differentially expressed genes (DEG), displaying fold change of genes (log_2_ scale) and *P* values (− log_10_ scale) between cuprizone (CPZ) treatment vs. control across 4 groups; wild-type, *Trem2*^R47H NSS^, *Trem2*^R47H CSS^, and *Trem2* KO (FC = 0.5, FDR < 0.05). **b** Heatmap of selected DEG from *Trem2*^R47H NSS^ (FDR < 0.05 for CPZ vs. control) compared across mouse models (see color scheme in b). Asterisk denotes the group of interest, *Trem2*^R47H NSS^ on CPZ. **c**, **d** List of inflammation DEG upregulated in CPZ compared to control diet that are found to be either (c) *Trem2*-dependent (upregulated in all groups but *Trem2* KO) or (d) *Trem2*-independent (upregulated in all groups). **e** List of uniquely downregulated DEG only found in *Trem2*^R47H NSS^. **f** List of myelination-related genes seen down-regulated in CPZ-treated mice across all groups. **g** Subset of modulerait relationship heatmap by PyWGCNA on wild-type, *Trem2*^R47H NSS^, *Trem2*^R47H CSS^, and *Trem2* KO associated with CPZ treatment**.** Color corresponding to correlation (red denotes positive correlation; blue denotes negative correlation) and the number in parenthesis shows relative significance of each correlation. Two modules (white and lightcoral modules) were chosen based on their significant correlation with CPZ treatment. **h**, **j** Barplots for the eigengene of the genes in the white and lightcoral modules, respectively. **i k** Gene ontology analysis of the genes in the white and lightcoral modules, respectively. *n* = 4–8. Data are represented as mean ± SEM. Two-way ANOVA followed by Tukey’s post hoc tests to examine biologically relevant interactions. Statistical significance is denoted by *p < 0.05, **p < 0.01, ***p < 0.001, ****p < 0.0001

Volcano plots of brain gene expression in cuprizone treated vs. control mice across the 4 groups reveal that animals in wild-type, *Trem2*^R47H NSS^, and *Trem2*^R47H CSS^ groups all show clear upregulation of inflammatory genes in response to cuprizone that is markedly suppressed in the *Trem2* KO group (Fig. 2a). We selected differentially expressed genes (DEGs) from the *Trem2*^R47H NSS^ mice (FDR < 0.05 for cuprizone vs. control) and created a heatmap to compare the response to that in the other 3 groups (Fig. 2b). Upregulated genes are all associated with inflammation and are mostly shared with the wild-type and *Trem2*^R47H CSS^ groups and include DAM genes such as *Apoe*, *Clec7a*, and *Itgax* (Fig. 2c), consistent with CD11c histology (Fig. 1k). Some inflammatory genes are also significantly upregulated in *Trem2* KO mice suggesting their induction is *Trem2* independent and includes more classical inflammation related genes such as *C1qa*, *Hmox1*, *Tyrobp*, and *Trem2* itself (Fig. 2d and Supplemental Fig. 3b). Downregulated genes in *Trem2*^R47H NSS^ include a unique set not altered in wild-type, *Trem2*^R47H CSS^, or *Trem2* KO groups, and are associated with dopaminergic signaling in the striatum (Fig. 2e). Shared downregulated genes are associated with myelin and oligodendrocytes, including *Cldn11*, *Mal*, *Mobp*, *Opalin*, and *Plp1* (Fig. 2f), suggesting that demyelination induced by cuprizone is not dependent upon the *Trem2*-dependent inflammation. Comparisons of DEGs between the three genotypes compared to wild-type in the absence of cuprizone treatment are shown as volcano plots (Supplemental Fig. 3c). Notably, *Trem2*^R47H CSS^ brains show more DEGs compared to wild-type mice than *Trem2*^R47H NSS^ (Supplemental Fig. 3d). Likewise, exploration of the number of DEGs induced by cuprizone in each of the four genotypes further revealed an extensive number of genes induced in the *Trem2*^R47H CSS^ brains that are not induced by cuprizone in wild-type, *Trem2*^R47H NSS^ or *Trem2* KO brains (Supplemental Fig. 3e; shown as a heatmap in Supplemental Fig. 3f).

To further explore gene expression changes across the groups, we analyzed functional networks of correlated genes (weighted correlation gene network analysis (WGCNA)) and identified two modules (White and Lightcoral) associated with cuprizone treatment (Fig. 2g). White module eigengene values were increased with cuprizone to similar extents in *Trem2*^R47H NSS^ and *Trem2*^R47H CSS^ mice, but significantly decreased in *Trem2* KO compared to wild-type mice (Fig. 2h), with gene ontology being associated with inflammation (Fig. 2i). Lightcoral module eigengene values were reduced in both *Trem2*^R47H NSS^ and *Trem2* KO mice with cuprizone but not in wild-type or *Trem2*^R47H CSS^ mice (Fig. 2j), and gene ontology associated with myelination and lipid metabolism (Fig. 2k), suggesting that the presence of *Trem2*^R47H NSS^ induces the appropriate inflammatory response to demyelination yet exacerbates its effects on myelination gene expression compared to wild-type *Trem2*. As myelin acts as a TREM2 ligand, inducing similar gene expression changes in microglia as exposure to Aβ plaques, we then compared all modules to AMP-AD identified modules that define gene expression changes in AD [50], for significant gene overlap (Supplemental Fig. 4). Notably, the White module (i.e., microglia/inflammation) strongly overlaps with the immune response and cytokine signaling AMP-AD modules, while the Lightcoral module (i.e., myelin and lipid metabolism related) strongly overlaps with the myelination AMP-AD modules. In addition to these two modules, the Lightgray module has significant overlap with the neuronal-related AMP-AD modules and is significantly inversely correlated to gene expression in *Trem2*^R47H NSS^, but not *Trem2*^R47H CSS^ or *Trem2* KO brains (Fig. 2g). Collectively, these results show that *Trem2*^R47H NSS^ mice show appropriate expression of *Trem2* transcripts, and that the *Trem2*^R47H NSS^ allele does not function as a null allele as assessed by cuprizone challenge.

### The *Trem2*^R47H NSS^ allele produces disease stage dependent effects on plaque density, size, and intensity

**Fig. 3:**
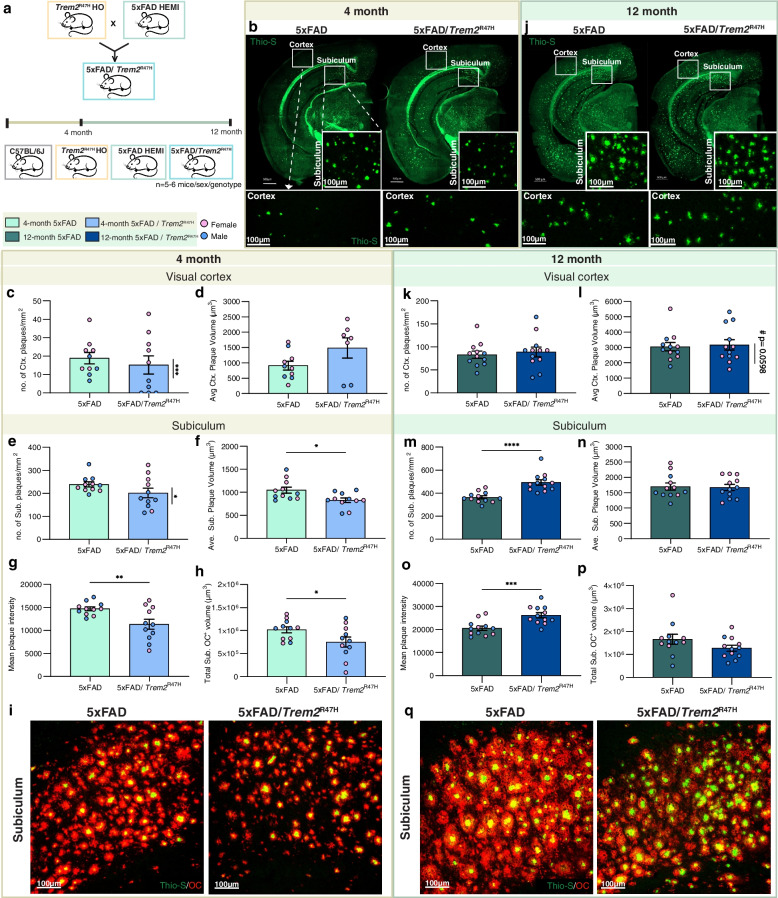
Initial sex-dependent amyloid plaque development in 5xFAD/*Trem2*^R47H^ mice. **a** Schematic showing mouse groups and study design. **b**, **j** Representative hemispheric coronal brain images of (b) 4-month-old and (j) 12-month-old 5xFAD and 5xFAD/*Trem2*^R47H^ stained for dense-core plaques using Thioflavin-S (green) with insets of higher magnification images of the (c, k) visual cortex and (e, m) subiculum. Scale bar = 500 µm. **c**, **d** In 4-month-old mice, there is a sex-dependent difference in density of Thio-S^+^ dense-core plaque burden within 5xFAD/*Trem2*^R47H^ in the visual cortex (c**,** plaque density; d**,** plaque volume; vertical significance bars). **d**, **f** Average plaque volumes showed no difference in (d) the visual cortex but decreased in (f) the subiculum of 5xFAD/*Trem2*^*R47H*^ compared to 5xFAD. **g** Reduced mean plaque intensity in 5xFAD/*Trem2*^R47H^ compared to 5xFAD at 4-months of age. **h** Quantification of total volume of OC^+^ diffused plaques in the subiculum shows less diffused plaques in 5xFAD/*Trem2*^R47H^ compared to 5xFAD at 4 months. **i**, **q** Representative confocal images of immunostained subiculum for OC (red) and Thio-S (green) at (i) 4-months and (q)12-months. **k**, **m** In 12-month old mice, there was no difference in density of dense-core plaques in the visual cortex of 5xFAD and 5xFAD/*Trem2*^R47H^ mice (k) but an increase in the subiculum (m). **l**, **n** No difference was observed in average plaque volume in (l) the cortex and (n) subiculum although there was a trend towards male 5xFAD/*Trem2*^*R47H*^ mice having larger plaques than females in (l) the cortex. **o** At 12 months, total mean plaque intensity is greater in 5xFAD/*Trem2*^R47H^ compared to 5xFAD. **p** Total volume of OC^+^ diffused plaques in the subiculum show lesson difference in diffuse plaques in 5xFAD/*Trem2*^R47H^. *n* = 10–12. Data are represented as mean ± SEM. Student’s t-test. Statistical significance is denoted by *p < 0.05, **p < 0.01, ***p < 0.001, ****p < 0.0001. Statistical trends are given by # 0.05 < *p* < 0.1

To investigate the contributions of *Trem2*^R47H^ to the pathogenesis of AD, we bred *Trem2*^R47H NSS^ mice with 5xFAD mice on a congenic B6J background to generate 4 groups: (i) wild-type, (ii) homozygous *Trem2*^R47H NSS^, (iii) hemizygous 5xFAD, and (iv) hemizygous 5xFAD; homozygous *Trem2*^R47H NSS^. We used *Trem2*^R47H NSS^ homozygotes rather than the heterozygous *TREM2*^R47H^ allele found in human AD individuals, to exacerbate phenotypes associated with the R47H mutation. Hereafter, for simplicity we refer to the *Trem2*^R47H NSS^ genotype as *Trem2*^R47H^. Mice were aged to 4 and 12 months and analyzed. Four-month-old 5xFAD mice are in their rapid plaque growth stage, which then plateaus throughout the brain by ~ 8–12 months depending on brain region [37]. While plaque densities are similar for both 5xFAD and 5xFAD/*Trem2*^R47H^ 4-month-old mice when collapsed cross the sexes (Fig. 3c, e), homozygosity for Trem2*R47H induces a robust sex difference in the manifestation of Thio-S^+^ plaques in both the cortex and subiculum, with lower plaque load in male 5xFAD/*Trem2*^R47H^ vs. 5xFAD mice (Supplemental Fig. 5b, n). Similar sex differences for plaque densities have been reported for *Trem2* KO mice crossed with 5xFAD mice [51]. Furthermore, female 5xFAD/*Trem2*^R47H^ mice exhibit increased plaque volume in the cortex compared to the age-matched 5xFAD mice (Supplemental Fig. 5e). Plaques in the subiculum of 4-month-old 5xFAD/*Trem2*^R47H^ mice are smaller with reduced mean plaque intensity compared to 5xFAD mice (Fig. 3f, g). Differences in the effects of *Trem2*^R47H^ on plaque volume between the cortex (where they are larger) and subiculum (where they are smaller) may be disease stage dependent as pathology begins earlier in the subiculum than the cortex, where at this timepoint plaques are only just beginning to form. Immunostaining for Aβ fibrillary oligomers using the conformation specific antibody OC, revealed less OC^+^ material, which includes more diffuse material in addition to the dense cores, in 5xFAD/*Trem2*^R47H^ compared to 5xFAD mice in the subiculum at 4 months (Fig. 3h, i). Quantification of Thio-S^+^ plaques in the visual cortex at the 12-month time point shows no differences (Fig. 3k-l), but there is a trend towards increased plaque volume in male compared to female 5xFAD/*Trem2*^R47H^ mice (Supplemental Fig. 5h). In the subiculum, 5xFAD/*Trem2*^R47H^ mice have higher plaque counts with comparable sizes (Fig. 3m-n). However, increased mean Thio-S^+^ plaque intensity in 5xFAD/*Trem2*^R47H^ mice is seen, with no difference in OC^+^ plaques (Fig. 3o-q). In the cortex, however, there is no difference in either mean plaque intensity or total OC^+^ volume at both 4- and 12-months (Supplemental Fig. 6).

**Fig. 4:**
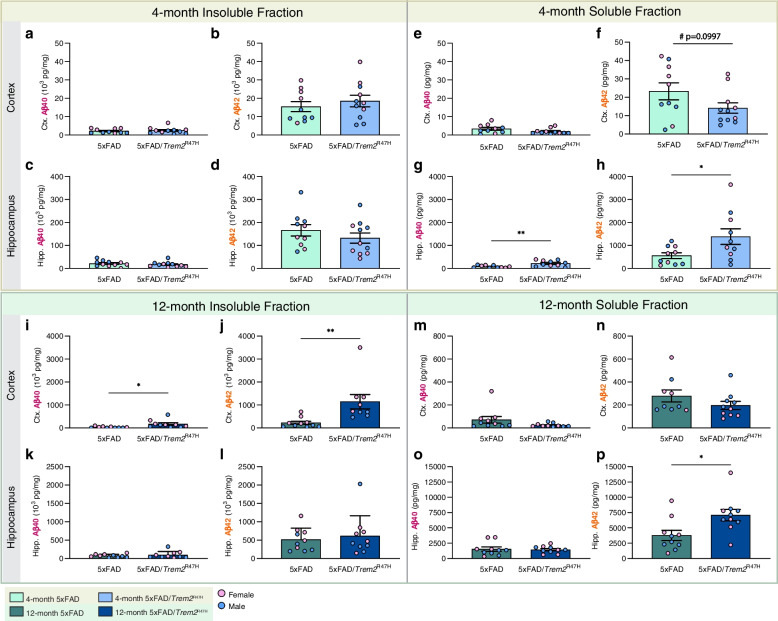
Quantification of insoluble and soluble Aβ in micro-dissected hippocampi and cortices. **a**-**d** No difference in insoluble Aβ40 and Aβ42 in hippocampus in 4-month-old 5xFAD/ *Trem2*^*R47H*^ vs 5xFAD mice. **e**–**h** In the soluble fraction, there is no difference in cortical Aβ40 (e), but a trending decrease in Aβ42 level was observed in the cortical fraction of 4-month-old 5xFAD/ *Trem2*^*R47H*^ vs 5xFAD animals (f, *p* = 0.0997). Increases in (g) Aβ40 and (h) Aβ42 are observed in hippocampal fraction of 5xFAD/ *Trem2*^*R47H*^ compared to 5xFAD. **i**-**l** At 12-months, insoluble cortical (i) Aβ40 and (j) Aβ42 are increased in 5xFAD/*Trem2*^R47H^ compare to 5xFAD while no difference was observed in hippocampal fraction (k, l). **m**-**p** No difference in soluble fraction except for an increase in hippocampal Aβ42 in 5xFAD/*Trem2*^R47H^ (p). Data are represented as mean ± SEM. Student’s t-test. Statistical significance is denoted by *p < 0.05, **p < 0.01, ***p < 0.001, ****p < 0.0001. Statistical trends are given by # 0.05 < *p* < 0.1

We quantified Aβ40 and Aβ42 in detergent soluble and insoluble fractions from microdissected hippocampi and cortices. At 4 months of age, increased soluble Aβ40 and 42 are found in the 5xFAD/*Trem2*^R47H^ hippocampus (Fig. 4h) and a trending reduction in soluble Aβ42 in the cortex (Fig. 4f), but no difference in the insoluble fraction in either brain region (Fig. 4a-d), compared to 5xFAD mice. By 12 months of age, Aβ is increased in both brain regions and fractions, with increased soluble Aβ42 in the hippocampus and increased insoluble Aβ40 and 42 in the cortex of 5xFAD/*Trem2*^R47H^ mice (Fig. 4i-p) compared to 5xFAD mice. Collectively, these results show that *Trem2*^R47H^ impacts the level of both plaque and Aβ in a brain region and age-specific manner.

### Initial impairment in microglial-plaque interactions is lost with age/disease progression

**Fig. 5:**
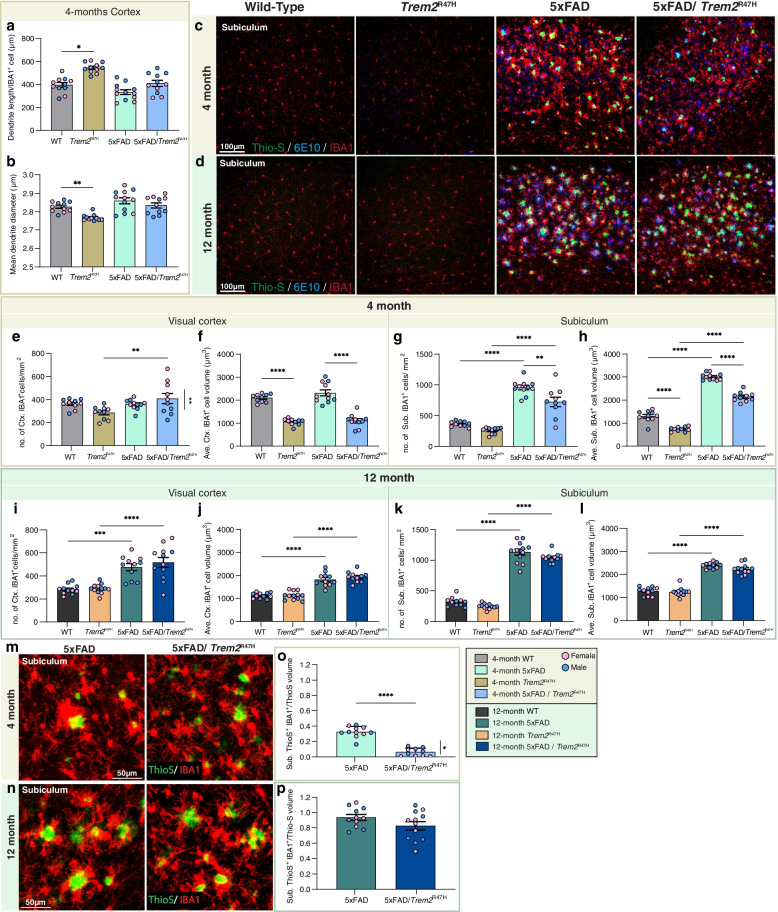
Age/disease-dependent impairment of plaque-microglia interaction driven by *Trem2*^R47H^. **a**, **b** Quantification of cortical microglial morphology of wild-type (WT), *Trem2*^R47H^, 5xFAD, and 5xFAD/*Trem2*^R47H^ revealed (a) increased dendrite length per IBA1^+^ cell in *Trem2*^R47H^ compared to WT but (b) decreased average dendrite diameter. **c**, **d** Subiculum—representative confocal images from wild-type, *Trem2*^R47H^, 5xFAD, and 5xFAD/*Trem2*^R47H^ mice at (c) 4- and (d) 12-months old stained with Thio-S for dense-core plaques (green), immunolabeled with 6E10 for diffused plaque (blue), and IBA1 for microglia (red). **e**–**h** Quantification of IBA1^+^ cell density and average volume in the (e, f) visual cortex and (g, h) subiculum at 4-months of age**.** In the cortex (e**)** a sex-dependent increase in microglia number and (f) a decrease in average microglial volume in the presence of *Trem2*^R47H^ are found. **g**, **h** In the subiculum, a decrease in both microglial density (g) and volume (h) is observed in 5xFAD/*Trem2*^R47H^ compared to 5xFAD. **i** -**l** IBA1^+^ cell density and average volume in the (i, j) visual cortex and (k, l) subiculum at 12-months-old. **m**–**n** Representative images of Thio-S (green) and IBA1 (red) colocalization in the subiculum at (m) 4-months and (n) 12-months old. **o**, **p** Quantification of percent colocalized volume of Thio-S^+^ and IBA1^+^ cell normalized to total Thio-S volume per field of view in the subiculum revealed decreased plaque-microglia interaction in 5xFAD/*Trem2*^R47H^ at (o) 4-months with sex-differences but not at (p) 12-months old. n = 10–12. Data are represented as mean ± SEM. Two-way ANOVA followed by Tukey’s post hoc tests to examine biologically relevant interactions. Statistical significance is denoted by *p < 0.05, **p < 0.01, ***p < 0.001, ****p < 0.0001

Given the expression of *Trem2* by microglia in the brain, we looked for changes in microglial densities and morphologies in both a non-pathologic and early pathologic (i.e., cortical regions of 5xFAD mice without overt dense core plaque load) state. Morphological analyses of cortical IBA1^+^ microglia showed increased process length but decreased diameter in *Trem2*^R47H^ compared to WT mice, while these differences were less apparent in the 5xFAD hemizygous background (Fig. 5a, b; images and sample analyses shown in Supplemental Fig. 7). We next imaged microglia and plaques in the visual cortex and subiculum (Fig. 5c, d). At 4-months old, as expected, the sex difference observed in plaque load of 5xFAD/*Trem2*^R47H^ mice is reflected in cortical microglial density with females showing higher microglia and plaque densities (Fig. 5e; data separated by sex included in Supplemental Fig. 8). In the subiculum, where Thio-S^+^ plaques are abundant, IBA1^+^ microglial density and size are increased in 5xFAD and 5xFAD/*Trem2*^R47H^ compared to wild-type mice (Fig. 5g, h). However, IBA1^+^ cell densities and volumes of 5xFAD/*Trem2*^R47H^ are lower than 5xFAD mice (Fig. 5g, h). Notably, in both brain regions, *Trem2*^R47H^ mice exhibit lower IBA1^+^ cell volume compared to WT, indicating that the *Trem2*^R47H^ variant elicits a plaque-independent effect on microglia morphology (Fig. 5f, h). A lack of plaque-microglia interaction is found in 5xFAD/*Trem2*^R47H^ mice as shown through quantification of Thio-S and IBA1 colocalization in the subiculum, with a further impairment in female 5xFAD/*Trem2*^R47H^ mice compared to males (Fig. 5o). However, the decreased microglia volume in *Trem2*^R47H^ mice and 5xFAD/*Trem2*^R47H^ compared to WT and 5xFAD, respectively, is absent at 12-month (Fig. 5 j, l)*,* as is the impairment in plaque-microglia interaction, suggesting age/disease-dependent changes in the *Trem2*^R47H^ variant effect on microglia morphology and function (Fig. 5m-p).

We quantified astrocytes in the visual cortex and subiculum and found that the *Trem2*^R47H^ variant has minimal effects on the astrocytic response to plaques at both 4- and 12-months (Supplemental Fig. 9).

### *Trem2*^R47H^ increases neuronal injury in response to plaques

**Fig. 6:**
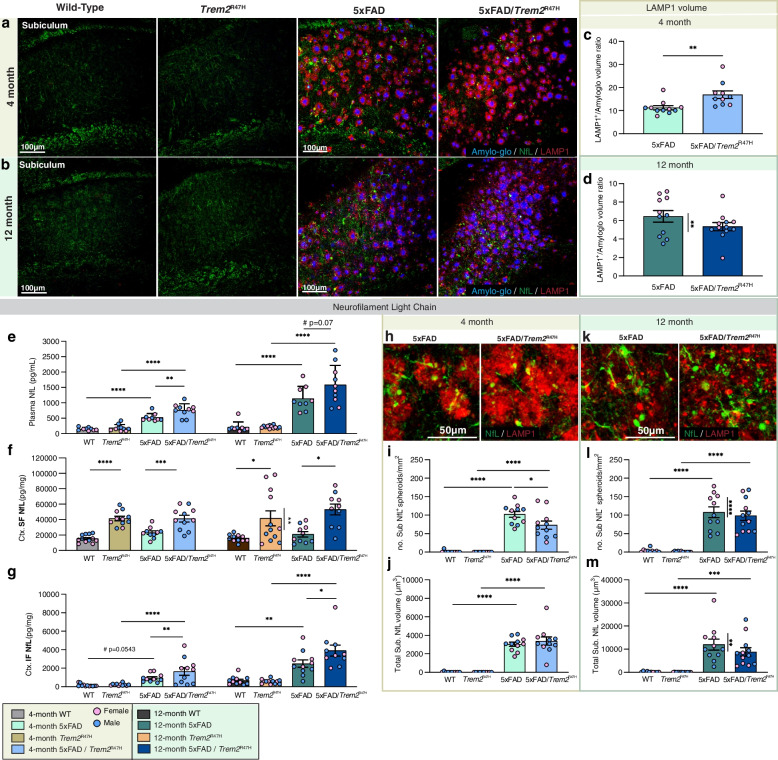
*Trem2*^R47H^ induces age/disease-dependent dystrophic neurites and axonal damage. **a**, **b** Representative confocal images of subiculum in (a) 4- and (b) 12-month-old wild-type, *Trem2*^R47H^, 5xFAD, and 5xFAD/*Trem2*^R47H^ mice stained with Amylo-Glo for dense-core plaques (blue) and immunolabeled for neurofilament light chain (NfL, green) and LAMP1 (red) for dystrophic neurites. **c**, **d** Quantification of subiculum LAMP1 volume normalized to Amylo-Glo volume shows increased dystrophic neurites at (c) 4-month but not at (d) 12-month with sex-difference in 5xFAD indicated. **e**–**g** Measurement of NfL in (e) plasma, (f) soluble fraction reveals consistent increase in NfL level in 5xFAD/*Trem2*^R47H^ compared to 5xFAD at both 4- and 12-months of age, and (g) cortical insoluble fraction. **h**, **k** Representative higher magnification images of immunolabeled NfL spheroids (green) colocalized with LAMP1 (red) in the subiculum of (h) 4-month-old and (k) 12-month-old 5xFAD and 5xFAD/*Trem2*^R47H^ mice. **i**, **j** Reduced density of NfL^+^ spheroids (i) but no change in spheroid volume (j) in subiculum of 5xFAD/*Trem2*^R47H^ compared to 5xFAD mice at 4-months of age. **l**, **m** No difference in number (l) or volume (m) of NfL^+^ spheroids between 5 and 5xFAD/*Trem2*^R47H^ despite a sex-difference in 5xFAD at 12-months of age. *n* = 10–12. Data are represented as mean ± SEM. Two-way ANOVA followed by Tukey’s post hoc tests to examine biologically relevant interactions. Statistical significance is denoted by *p < 0.05, **p < 0.01, ***p < 0.001, ****p < 0.0001

Microglia form a protective barrier around plaques while contributing to their compaction and growth [21]. Given the initial impairment in microglial-plaque interactions in 5xFAD/*Trem2*^R47H^ mice we next explored the halo of dystrophic neurites that develops around dense-core plaques [52, 53] which can be visualized with lysosomal-associated membrane protein 1 (LAMP1, Fig. 6a, b). Normalization of LAMP1 volume to plaque volume reveals an increase in dystrophic neurites per plaque area in 5xFAD/*Trem2*^R47H^ mice at 4 months of age (Fig. 6c). Consistent with the restoration of microglial-plaque interactions at 12 months of age, no difference in dystrophic neurites per plaque is seen between 5xFAD/*Trem2*^R47H^ and 5xFAD mice at this age (Fig. 6d). Twelve-month-old 5xFAD females have more dystrophic neurites than male (Fig. 6d). Interestingly, LAMP1^+^ dystrophic neurites exhibit a more dissipated morphology at 12-month compared to 4-month, consistent with disrupted axonal transport at later age/disease stages [54].

Neurofilament light chain (NfL) is emerging as a clinically useful plasma biomarker for damage occurring in the brain, including in AD where it tracks with cortical thinning and cognitive decline [55–57], while in mouse models of AD it correlates with plaque load [38]. We measured plasma NfL as a surrogate marker of brain damage and found it increased in 5xFAD mice compared to WT mice at 4 months of age, with further increase at 12-months of age, consistent with plaque load (Fig. 6e). The *Trem2*^R47H^ variant increased plasma NfL in 5xFAD at both ages (Fig. 6e), in line with the hypothesis that TREM2 dysfunction exacerbates neuronal damage. We also measured NfL levels in the detergent soluble and insoluble fractions of microdissected cortices (Fig. 6f, g). NfL in the soluble fraction is not increased in 5xFAD mice compared to WT at either 4 or 12 months of age but is elevated by the presence of *Trem2*^R47H^ (Fig. 6f). NfL in the insoluble fraction aligns with levels in the plasma, with increases seen in 5xFAD compared to wild-type mice at 4 months and further increases at 12 months (Fig. 6g). As with plasma, NfL levels further trend higher in 5xFAD/*Trem2*^R47H^ mice. Thus, the presence of *Trem2*^R47H^ induces changes in NfL, and further exacerbates plaque-induced increases in both detergent insoluble and plasma NfL. To identify the cellular source of NfL, we immunostained for dystrophic neurites (LAMP1) and NfL. Large spherical structures of NfL are seen in the vicinity of plaques and are absent from wild-type and *Trem2*^R47H^ mice, where staining is observed only in axonal fibers (Fig. 6a, b). Similar bead-like NfL^+^ spheroids were reported in ischemia-affected human and mouse tissues as sign of axonal damage [58]. Notably, these NfL spheroids colocalize with dystrophic neurites associated with plaques (Fig. 6h, k). Quantification of NfL^+^ structures showed a decreased spheroid number in 5xFAD/*Trem2*^R47H^ compared to 5xFAD, while having similar size at 4-month (Fig. 6i, j). At 12-month, no difference in NfL^+^ spheroid number or size is observed between 5xFAD and 5xFAD/*Trem2*^R47H^ brains. However, in 5xFAD, there is a sex difference with females having more NfL compared to males, which is also observed in LAMP1^+^ dystrophic neurite amount (Fig. 6d; data separated by sex included in Supplemental Fig. 10). Collectively, these findings reveal associations between plaques and dystrophic neurites with NfL accumulation and its transition to the insoluble fraction and plasma NfL.

### Trem2^R47H^ protects against plaque-induced LTP and synaptic deficits

**Fig. 7:**
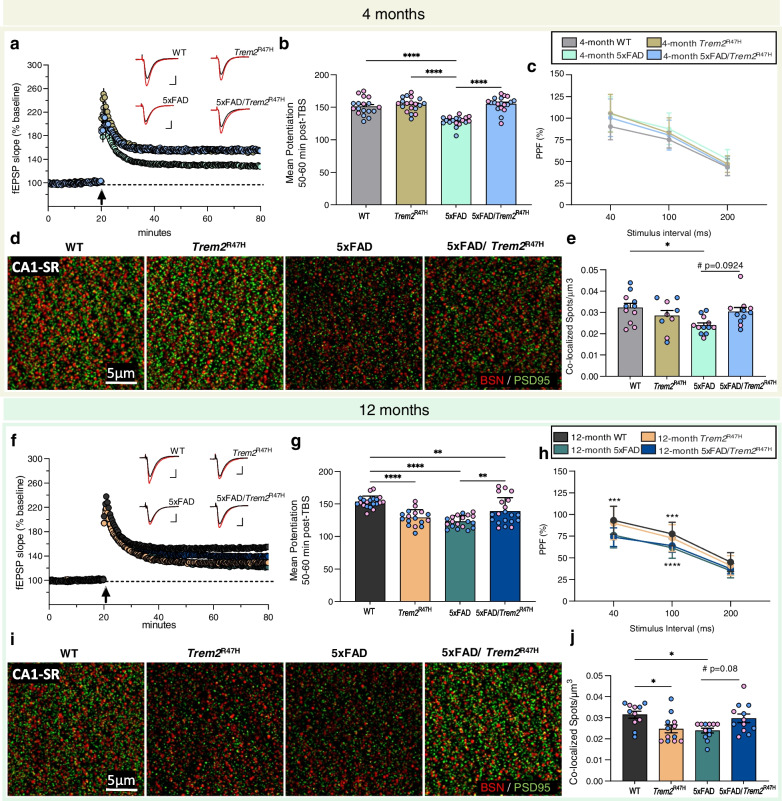
Effect of *Trem2*^R47H^ on age-dependent LTP deficit. **a** Time course of fEPSP slope (as percentage of baseline) following theta burst stimulation (TBS, white arrow at t = 20 min) of slices from 4-month-old WT, *Trem2*^R47H^, 5xFAD, and 5xFAD/*Trem2*^R47H^ mice showing impaired LTP in 5xFAD but not 5xFAD/*Trem2*^R47H^. Insets show field synaptic responses collected during baseline (black line) and 1 h after TBS (red line). Scale: 1 mV/5 ms. **b** Mean potentiation (± SEM) during the last 10 min of recording in slices from 4-month-old mice shows reduction in 5xFAD but not 5xFAD/*Trem2*^R47H^ mice. **c** Paired-pulse facilitation (PPF). At 4 months old, no significant difference was observed between groups at any of the three intervals tested. **d** Representative super-resolution images at 63X objective of CA1-SR from 4-month-old WT, *Trem2*^R47H^, 5xFAD, and 5xFAD/*Trem2*^R47H^ mice immunolabeled with Bassoon for presynaptic elements (BSN, red) and PSD-95 for postsynaptic elements (green). **e** Quantification of Bassoon^+^ and PSD-95^+^ spots per µm^3^ showed a decrease in colocalized synaptic puncta in 5xFAD. **f** Time course of fEPSP slope following theta burst of slices from 12-month-old WT, *Trem2*^R47H^, 5xFAD, and 5xFAD/*Trem2*^R47H^ mice show impaired LTP in *Trem2*^R47H^, 5xFAD and partial impairment in 5xFAD/*Trem2*^R47H^. **g** Mean potentiation (± SEM) during the last 10 min of recording in slices from 12-month-old mice shows reduction in *Trem2*^R47H^, 5xFAD, 5xFAD/*Trem2*^R47H^ but a partial rescue in 5xFAD/*Trem2*^R47H^ compared to 5xFAD. **h** Significant group effect was found in PPF at 40 ms (WT vs 5xFAD and WT vs 5xFAD/*Trem2*^R47H^; p = 0.0002, p < 0.0001 respectively) and 100 ms stimulus intervals (WT vs 5xFAD and WT vs 5xFAD/*Trem2*^R47H^; p = 0.0005, p = 0.0046 respectively). **i.** 63X representative super-resolution images of CA1-SR from 12-month-old WT, *Trem2*^R47H^, 5xFAD, and 5xFAD/*Trem2*^R47H^ mice for Bassoon (red) and PSD-95 (green). **j** Quantification of Bassoon^+^ and PSD-95^+^ colocalization showed decrease in colocalized synaptic puncta in *Trem2*^R47H^ and 5xFAD compared to WT with a trending increase in 5xFAD/*Trem2*^R47H^ compared to 5xFAD. n = 5–6 mice/sex/genotype. LTP: n = 8–10 slices/sex/genotype. Data are represented as mean ± SEM. Two-way ANOVA followed by Tukey’s post hoc tests to examine biologically relevant interactions. Statistical significance is denoted by *p < 0.05, **p < 0.01, ***p < 0.001, ****p < 0.0001

Due to the increased dystrophic neurites induced by the R47H variant, we investigated short- and long-term synaptic plasticity in WT, *Trem2*^R47H^, 5xFAD, and 5xFAD/*Trem2*^R47H^ hippocampi via theta burst-induced pair-pulse facilitation (PPF) and long-term potentiation (LTP) in acute hippocampal slices. Consistent with our previous findings [37], 5xFAD have impaired LTP at 4 months of age. Remarkably, this impairment is suppressed by the presence of *Trem2*^R47H^ (Fig. 7a, b), with no change in PPF (Fig. 7c). Consistent with a lack of LTP impairment in 5xFAD/*Trem2*^R47H^ mice, immunostaining of pre-synaptic (Bassoon) and post-synaptic (PSD-95) elements at the CA1 stratum radiatum (CA1-SR) using super-resolution structured illumination microscopy revealed a decrease in co-localized pre- and post-synaptic puncta in 5xFAD animals compared to wild-type mice, which is not seen in 5xFAD/*Trem2*^R47H^ mice compared to *Trem2*^R47H^ mice (Fig. 7d, e). At 12-months of age, LTP deficits are also seen in *Trem2*^R47H^, as well as 5xFAD, and, to a lesser degree, 5xFAD/*Trem2*^R47H^, compared to WT animals (Fig. 7f, g). Notably, PPF responses in 12-month-old mice show a decrease in presynaptic plasticity in both 5xFAD and 5xFAD/*Trem2*^R47H^ compared to their non-5xFAD controls at 40 ms and 100 ms stimulus intervals, although, the effect in 5xFAD/*Trem2*^R47H^ is absent at 200 ms interval (Fig. 7h). Quantification of CA1-SR synapses at 12 months recapitulates the LTP results—reduction of synaptic co-localization in *Trem2*^R47H^ and 5xFAD compared to WT with a trending rescue in the 5xFAD/*Trem2*^R47H^ vs 5xFAD (Fig. 7i, j). Postsynaptic elements are also decreased in 12-month-old *Trem2*^R47H^ compared to WT, along with an increase in 5xFAD/*Trem2*^R47H^ vs 5xFAD, which reflect the observed fEPSP (Fig. 7g) and mean potentiation data (Supplementary Fig. 11 l). Lastly, basic synaptic transmission was assessed by examining the input–output relationship of the synaptic response in all four groups of mice (Supplemental Fig. 11). As stimulus intensities increased, the amplitude of the fiber volley is significantly reduced in slices from 4 months 5xFAD, *Trem2*^R47H^ and 5xFAD/*Trem2*^R47H^ mice relative to WT controls indicating reductions in synaptic transmission (Supplemental Fig. 11a-c). By 12 months of age, the amplitude of the fiber volley over a range of stimulus intensities is indistinguishable from control WT. However, a slight reduction in the slope of the fEPSP is measured in all three experimental groups with respect to controls (Supplemental Fig. 11 g-i). These results suggest that 5xFAD mice may have a reduction in afferent activation, but also reveals deficits in synaptic transmission in *Trem2*^R47H^ and 5xFAD/*Trem2*^R47H^ mice that are not fully explained by long-term plasticity changes.

### *Trem2*^R47H^ initially suppresses then enhances neuroinflammation with age/disease progression, including production of a unique interferon signature

**Fig. 8:**
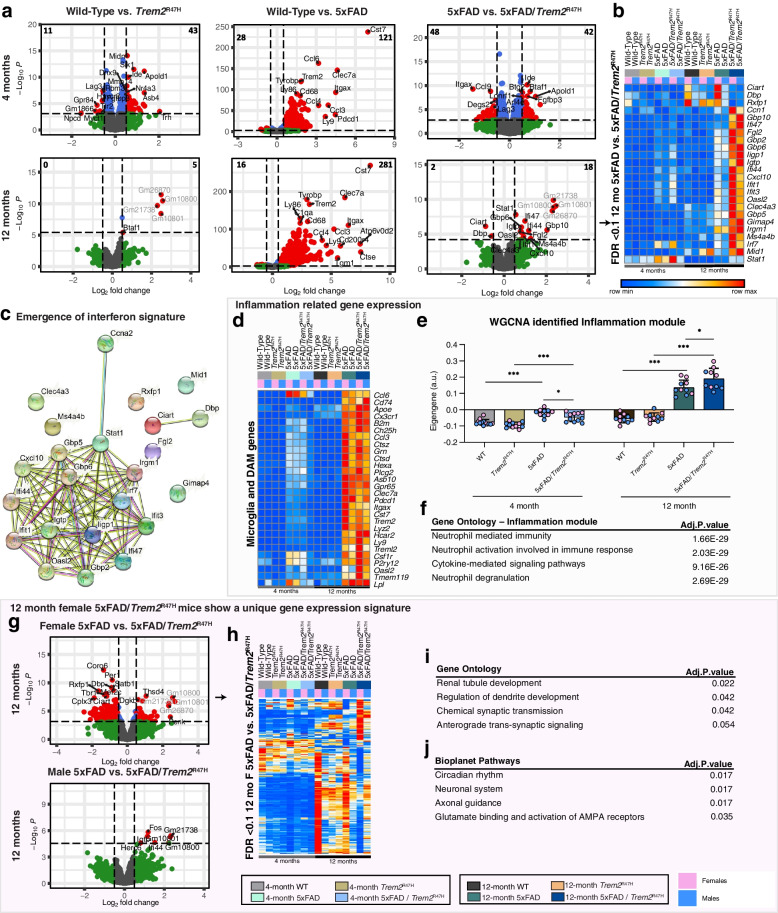
*Trem2*^R47H^ initially suppresses then enhances neuroinflammation with age/disease progression. **a** Volcano plot of DEGs, displaying fold change of gene expression (log_2_ scale) and *P* values (− log_10_ scale) at 4- (upper panel) and 12-months old (lower panel) between *Trem2*^R47H^ vs wild-type, 5xFAD vs wild-type, and 5xFAD/*Trem2*^R47H^ vs 5xFAD. **b** Heatmap of selected DEGs in 5xFAD/*Trem2*^R47H^ vs 5xFAD at 12-months old, showing the list of uniquely upregulated genes only in 5xFAD/*Trem2*^R47H^ highlighting multiple interferon-related genes (FDR < 0.1). In second row of heatmap, pink boxes denote females while blue boxes denote males. **c** Gene network map shows connections of DEGs between 12-month-old 5xFAD/*Trem2*^R47H^ and 5xFAD mice. **d** Heatmap generated from selected DEGs in the inflammation (Darkgrey) module. **e** Eigengene of the Darkgrey module plotted as bar graphs for WT, *Trem2*^R47H^, 5xFAD and 5xFAD/*Trem2*^R47H^ at 4- and 12-month timepoints. **f** Gene ontology of the Darkgrey module shows increased neutrophil responses and pathways. **g**, **h** Volcano plot of DEGs, displaying fold change of gene expression (log_2_ scale) and *P* values (− log_10_ scale) of (g) female and (h) male 5xFAD/*Trem2*^R47H^ vs 5xFAD at 12-months old. **i** Heatmap generated from DEGs between 5xFAD/*Trem2*^R47H^ vs 5xFAD vs females at 12 months. **i** Gene oncology of the DEGs between 5xFAD/*Trem2*^R47H^ vs 5xFAD females at 12 months. **j** Bioplanet pathways associated with the DEGs between 5xFAD/*Trem2*^R47H^ vs 5xFAD females at 12 months. Eigengene bar plots—*n* = 10–15. Data are represented as mean ± SEM. Two-way ANOVA followed by Tukey’s post hoc tests to examine biologically relevant interactions. Statistical significance is denoted by *p < 0.05, **p < 0.01, ***p < 0.001, ****p < 0.0001

To assess gene expression changes with age, sex and genotype, we performed RNA-seq from microdissected hippocampi from 4- and 12-month-old WT, *Trem2*^R47H^, 5xFAD, and 5xFAD/*Trem2*^R47H^ mice. PCA plots show clustering of 4 vs 12-month samples accounting for most of the variance between samples (Supplemental Fig. 12a). Volcano plots illustrate changes in gene expression in *Trem2*^R47H^ relative to WT mice, 5xFAD relative to WT, and 5xFAD/ *Trem2*^R47H^ relative to 5xFAD mice at both 4- and 12- month timepoints (Fig. 8a). Differentially expressed genes (DEGs) in 5xFAD mice vs. WT mice at both ages are mostly upregulated genes and represent the strong inflammatory response seen in these mice, and include DAM genes such as *Cst7*, *Itgax*, *Clec7a*, as well as *Trem2*. Identified *Trem2*-dependent and -independent induction of inflammatory genes from the cuprizone experiments are shown as a heatmap in Supplemental Fig. 12b and c. DEGs between 5xFAD/*Trem2*^R47H^ vs 5xFAD mice at 12 months are plotted as a heatmap (Fig. 8b; FDR < 0.1, no FC cutoff). Notably, this subset consists of downregulated circadian related genes, *Ciart* and *Dbp,* compared to 5xFAD mice, but upregulation of many genes associated with interferon signaling, such as *Ifi47*, *Ifit1-3*, and *Gbp2*, *6*, and *7* (Fig. 8b). These interferon-related genes are also involved in a highly connected gene network (Fig. 8c).

To focus on the inflammatory response, we first selected homeostatic and disease-associated microglia genes and displayed them as a heatmap (Fig. 8d). This revealed a suppression of these genes in 4-month-old 5xFAD/*Trem2*^R47H^ hippocampi compared to 5xFAD hippocampi that was absent at 12 months of age. To further explore gene expression changes across the groups, we analyzed functional networks of correlated genes (WGCNA; module – trait relationships shown in Supplemental Fig. 12d) and identified a module containing inflammation associated genes (Fig. 8f; Darkgrey). We plotted eigengene values of this inflammation module for all groups (Fig. 8e), revealing an increase in 4-month-old 5xFAD mice compared to both WT and *Trem2*^R47H^ mice, corresponding to the inflammatory response to the plaques in those mice. Notably, no such increase in eigengene value is observed in 4-month-old 5xFAD/*Trem2*^R47H^ mice, showing a suppression of inflammation at the 4-month timepoint compared to 5xFAD mice, mirroring the LTP and synaptic deficits seen in these mice. However, by 12 months of age, robust increases in the inflammation module eigengene values are seen in both 5xFAD and 5xFAD/*Trem2*^R47H^ mice, with the presence of Trem2*R47H inducing an even higher increase (Fig. 8e). This further shows the initial suppression of inflammation induced by the Trem2*R47H variant dissipates with time/age/disease progression, consistent with the results of histology. We next compared all modules with the AMP-AD identified modules that define gene expression changes in AD brains [50]. The Darkgrey inflammatory module shares a significant and substantial overlap with the immune response and cytokine signaling modules (Supplemental Fig. 13a), which we established is enhanced in 12-month-old 5xFAD/*Trem2*^R47H^ compared to 5xFAD mice (Fig. 8e). The Lightgrey module has significant overlap with the AMP-AD neuronal systems modules and plotting of the eigengene values reveals a significant decrease in this module in 12-month-old 5xFAD/*Trem2*^R47H^ compared to 5xFAD mice (Supplemental Fig. 13b). Finally, the Snow module shows significant overlap with the AMP-AD myelination related modules and plotting of the eigengene values reveals a clear effect of *Trem2*^R47H^ on this module in 12-month-old mice, regardless of 5xFAD genotype (Supplemental Fig. 13d). Collectively, these results show that the presence of the *Trem2*^R47H^ variant induces a gene expression signature that better matches the spectrum of gene expression changes in human AD brains.

Given sex-specific transcriptional changes previously reported in *TREM2*^R47H^ variant-carrying AD patients and mice expressing *TREM2*^R47H^ cDNA crossed with PS19 mice, we explored gene expression changes in male and female mice comparing 5xFAD/*Trem2*^R47H^ with 5xFAD at 12 months [18]. Interestingly, 5xFAD/*Trem2*^R47H^ female mice show a unique gene expression signature compared to the age-matched 5xFAD mice (Fig. 8 g-h), which is not seen in males. Moreover, most of the genes downregulated in 5xFAD/*Trem2*^R47H^ females are highly upregulated in WT and less so in 5xFAD females, yet this effect is not observed in *Trem2*^R47H^, suggesting the presence of the *Trem2*^R47H^ variant dampens the expression of these genes in non-disease and further exacerbates in a disease model (Fig. 8h). Gene ontology analyses suggests these genes are involved in dendrite development, chemical synaptic transmission, and pathways such as circadian rhythm and axonal guidance (Fig. 8i-j).

Given the selective downregulation of *Itgax* and *CD74* at 4-months-of-age between 5xFAD/*Trem2*^R47H^ and 5xFAD mice (Fig. 8a), we performed immunofluorescence for both markers (Supplemental Fig. 14a, b), alongside microglia and plaques. CD11c (*Itgax*) and CD74 staining were seen in a subset of plaque-associated microglia, with greater numbers at 12- vs. 4-months of age. Concordant with gene expression data, CD11c and CD74 staining was reduced at 4-months of age (Supplemental Fig. 14e, f). However, at 12 months old, despite not reaching FDR of 0.1, *Itgax* TPM level, when examined independently and separate from other genes, shows a significant decrease in 5xFAD/*Trem2*^R47H^ compared to 5xFAD (Supplemental Fig. 14g), which is also observed via immunohistochemistry (Supplemental Fig. 14i). CD74 staining (Supplemental Fig. 14j) is also reflective of *CD74* TPM level at 12 months (Supplemental Fig. 14h) with no difference between 5xFAD and 5xFAD/*Trem2*^R47H^.

Collectively, these results show that the *Trem2*^R47H^ variant mediates an initial suppression of inflammation in response to plaques in 5xFAD mice, which is reversed and exacerbated with age/disease progression. Furthermore, the *Trem2*^R47H^ variant generates a unique interferon-related gene expression signature in 5xFAD mice.

## Discussion

The identification of coding sequence changes in *TREM2* that were strongly associated with increased risk for development of LOAD focused interest on both TREM2 function and microglia, which predominantly express TREM2 in the brain [13, 14]. Initial studies of *Trem2* KO mice found that TREM2 was necessary for the microglial reaction to plaques, as well as their transition to a “DAM” phenotype, characterized by the specific expression of genes such as *Cst7, Clec7a, Itgax*, and *Apoe* [59]. Furthermore, the absence of TREM2, coinciding with a lack of microglial reaction to plaques, appeared to paradoxically worsen disease progression [60–62]. Although these KO studies validated TREM2 as a key and central player for microglia in the pathogenesis of AD, they did not address how missense mutations in the protein could modify the risk of developing LOAD with age. Furthermore, a caveat with the interpretation of functional endpoints from AD models crossed with *Trem2* KO mice is that loss of TREM2 function (or its binding partner DAP12) in humans results in Nasu-Hakola disease, a white matter-targeting age-dependent neurodegenerative disease [63], suggesting that absence of TREM2 has detrimental effects on the brain in the absence of plaques.

The R47H missense mutation in *TREM2* is strongly and reproducibly linked to LOAD, and since its discovery, multiple studies have attempted to model this mutation in mice and rats. Several approaches have produced TREM2^R47H^ models, including via bacterial artificial chromosome (BAC) and via CRISPR/Cas9 technology. BAC transgenic mice expressing human common variant (CV) and R47H variants of *TREM2* have been crossed with *Trem2* KO/5xFAD mice, with the resultant phenotype phenocopying *Trem2* KO, therefore suggesting the *TREM2*^R47H^ variant is a loss-of-function allele of TREM2 [64]. As an extension of this approach, human *TREM2*^R47H^ and common variant cDNA has also been knocked into the mouse *Trem2* locus [18], such that animals express human *TREM2*, but without the full complement of regulatory machinery. Crossing of these mice with the PS19 mouse model of tauopathy found that the R47H variant exacerbates damage and inflammation and does not function as a loss of function variant [18], unlike similar crosses of PS19 mice with the human BAC *TREM2*^R47H^ model which actually protected against microglia activation and subsequent neurodegeneration [65], as did crosses with PS19 mice and *Trem2* KO mice [66, 67]. In addition to these humanized approaches, several *Trem2*^R47H^ mouse models have been generated via CRISPR/Cas9 technology and have reported similar findings as *Trem2* KO mice [34, 35]. However, it has since been reported that introduction of the R47H variant into mouse *Trem2* produced aberrant splicing, due to synonymous base changes co-introduced with repair templates to arrest Cas9 mediated cleavage, as we confirm here with long-read RNA-seq [35]. These models display significantly reduced *Trem2* expression [35], effectively making them hypomorphic alleles of *Trem2* that do not accurately reflect the human condition. Understanding how *TREM2* and its variants influence the development of LOAD is critical for our understanding of the disease. Therefore, the production of animal models that faithfully reproduce human gene function is crucial to accurately recapitulate the disease in rodents. To that end we, as part of the Model Organism Development and Evaluation for Late-onset Alzheimer’s Disease (MODEL-AD) consortium, embarked on the development of a *Trem2*^R47H^ mouse variant without the shortcomings of artificial cryptic splicing and reduced expression, by using a CRISPR/Cas9 approach utilizing a repair template based on a previous study [44]. The resultant *Trem2*^R47H NSS^ mouse has normal *Trem2* expression and normal splicing and is available without restriction to both academic and commercial entities (Jax stock: #034,036). Why the *Trem2*^R47H NSS^ allele and that generated by Xiang *et. al* do not appear to display cryptic splicing within exon 2 of *Trem2* is unclear (Supplemental Fig. 15) [35]. To investigate whether correct introduction of the R47H mutation results in a loss of function of TREM2, we utilized the cuprizone model of demyelination, as both myelin and Aβ act as TREM2 ligands, to assess the capacity of microglia to clear white matter debris and found that our model shared similar inflammation responses and pattern as wild-type mice, unlike cuprizone treated *Trem2* KO mice. However, despite normal induction of microglial evoked inflammation (including expression of “DAM” genes), we see evidence of increased oligodendrocyte gene expression loss in both *Trem2*^R47H NSS^ and *Trem2* KO mice, consistent with the notion that dysfunction or absence of TREM2 exacerbates damage in response to a suitable stimulus. Thus, the presence of the R47H variant with normal *Trem2* expression levels does not appear to function as a loss of function allele in response to a cuprizone challenge in terms of a microglial response but does phenocopy the exacerbated damage inferred by the null allele.

To give relevance to AD, we crossed *Trem2*^R47H NSS^ mice with the 5xFAD mouse model of amyloidosis and evaluated pathology and gene expression at 4 and 12 months of age. We identified a consistent sex difference in the initial appearance of plaques (4 months), with female *Trem2*^R47H^ mice producing more plaques compared to their male counterparts. Notably, a similar sex difference has also been observed in *Trem2* KO mice crossed with APP1/PS1 mice ([51]; females have more plaques) and human *TREM2*^R47H^ cDNA mice crossed to the PS19 tauopathy model (females have more inflammatory gene expression and spatial memory deficits), as well as transcriptomic analysis of R47H-carrying AD patients ([18]; females upregulating immune activation pathways while males upregulate metabolic and adenosine triphosphate pathway). No significant sex difference was observed by 12 months of age, but plaque density was increased by the presence of the R47H mutation, while both soluble and insoluble Aβ levels are also increased. Consistent with the R47H variant inferring a loss-of-function phenotype, the initial microglia-plaque interaction is impaired, resulting in smaller and less compacted plaques yet an increase in dystrophic neurites produced by those plaques, in line with prior data from *Trem2* KO mice [61, 62, 68]. However, these impairments between microglia and plaque interactions are absent by 12 months of age yet resulting in potential over-compaction of plaques, suggesting an overcompensation of microglial behavior with time.

Supporting these data, gene expression from microdissected hippocampi mirrors the initial impairments between microglia and plaques, with reduced expression of DAM genes such as *Itgax* and *Cd74*. However, by 12 months, when no impairments between microglia and plaques are seen, these DAM genes are no longer reduced with the exception of *Itgax,* where immunostaining suggests a decrease in protein level despite the reduction in *Itgax* expression not reaching FDR significance. The presence of the R47H variant induces a selective upregulation of interferon related genes such as *Ifi47*, *Ifit1-3*, and *Gbp2*, *6*, and *7*, which are all key players in pathogen response [69]. A similar interferon signal was also reported in R47H-carrying AD patients [18]. Furthermore, the WGCNA identified inflammatory module (Darkgrey) revealed increases in inflammation in the 5xFAD mice at 4 months of age were prevented by the presence of the R47H variant, but exceeding 5xFAD levels by 12 months of age. Thus, the R47H variant appears to confer age and disease specific effects on microglia. Of direct relevance and validating these results, similar findings have been shown in human AD tissue from *TREM2* variant carriers, in which microglial responses to pathology are suppressed in newly formed pathological areas but exacerbated in more advanced pathological brain areas [70]. A further module, Lightgrey, shows considerable overlap with neuronal modules identified in AD brains by AMP-AD [50], and is selectively decreased in 12-month-old 5xFAD/*Trem2*^R47H^ brains.

Given the fact that the R47H *TREM2* variant has been associated with several neurodegenerative diseases, we have also focused on how this variant may be more permissive of damage exerted on the brain by the relevant pathology, in this case plaques. As mentioned earlier, we see initial increases in dystrophic neurites induced by plaques in the presence of the R47H variant, supporting this notion. We further demonstrate increased plasma NfL, a reliable marker of brain injury that tracks with cortical thinning and cognitive decline in AD populations [55–57, 71], in the presence of plaques. We localized NfL in the brain to being associated with dystrophic neurites induced by plaques/microglia and found that NfL level in the brain insoluble fraction correlated with levels in the plasma. Collectively, these results highlight how the *Trem2*^R47H^ variant can induce greater damage on clinically relevant endpoints.

Noticeably, at 4 months, despite an increase in dystrophic neurites, the presence of the *Trem2*^R47H^ variant in a 5xFAD background protected against LTP deficits found in the 5xFAD mice, as well as protected against loss of synaptic puncta, consistent with reported effects of the R47H variant in the PS19 mouse model of tauopathy [65]. While the impaired microglia-plaque interactions induced by the *Trem2*^R47H^ variant promotes dystrophic neurites consistent with previous findings [21, 60], the blunted microglial function also prevents increases in inflammatory gene expression which could explain the lack of LTP deficits and synaptic loss at 4 months. Notably, by 12 months of age, *Trem2*^R47H NSS^ mice on a WT background also demonstrated robust impairments in LTP, as well as synaptic puncta loss. Similar results have been reported in a *Trem2*^R47H^ rat model, further implicating microglia and dysfunctional *Trem2* in effecting neuronal structure and function [72]. Moreover, at 12 months, although there is an overcompensation of microglial response with increased inflammatory gene profiles, there is still a lack of LTP and synaptic loss, suggesting that TREM2 and microglia have multiple actions that run counter to one another at different disease stages.

## Conclusions

A major objective of this study is to provide the scientific community with a reliable and well-characterized mouse *Trem2*^R47H^ knock-in model and highlight how this variant has age- and disease- dependent effects on microglia and neuropathology, mirroring reported human data. We demonstrate that *Trem2*^R47H^ has effects on the synaptic landscape with age, coinciding with robust impairments in LTP. Collectively, these results help to clarify prior data obtained from amyloidosis models crossed with *Trem2*^R47H^ mice with unintended hypomorph phenotypes [34, 35], and add to our understanding of how microglia and TREM2 contribute to the pathogenesis of AD.

## Supplementary Information

**Additional file 1.** .

**Additional file 2: Supplemental Figure 1. ** Long-read RNA-seq analysis of *Trem2* transcripts from whole brain of 15-wk old wild type, homozygous *Trem2*^R47H NSS^ and homozygous *Trem2*^R47H CSS^ mice. For each of the three genotypes, independent transcripts are shown in grey, aligned to the known five exons of *Trem2* (dark blue) with inferred intronic sequences in light blue. Total transcripts from one wild-type mouse are shown, and three from both *Trem2*^R47H NSS^ and *Trem2*^R47H CSS^ brains.  The top part of each figure shows a compressed view of the transcripts. For *Trem2*^R47H NSS^ and *Trem2*^R47H CSS^, the colored regions within exon 2 denote DNA bases that vary from the wildtype reference sequence. As reported [35], the *Trem2*^R47H CSS^ allele produces a significant number of transcripts that use a cryptic splice site within exon 2, which are not observed in either the wild type or *Trem2*^R47H NSS^ samples.

**Additional file 3: Supplemental Figure 2. ** Off-target site activity analysis for crRNA TMF1342 on mouse chromosome 17. a-k No difference was found in sequence between the C57BL/6J WT and *Trem2*^R47H NSS^alleles at each of the 11 potential off-target sites analyzed. The black underline denotes the crRNA target sequence while the blue underline denotes the NGG PAM site. l-m Long-read RNA-seq analysis of transcripts from one potential off-target locus (*Zfp945*) in brain shows no difference in the level of each isoform.  The green asterisk denotes the excluded exon compared to *Zfp945*-201.

**Additional file 4: Supplemental Figure 3. **Gene expression profile differences between *Trem2*^R47H NSS^, *Trem2*^R47H CSS^and *Trem2* KO at steady-state and following cuprizone challenge. a PCA plot of cuprizone (CPZ) dataset. b Heatmap generated from selected *Trem2*-independent upregulated inflammatory DEGs from Fig. 2b and d. c Volcano plot of DEG, displaying fold change of genes (log_2_ scale) and *P* values (−log_10_ scale) between *Trem2*^R47H NSS^, *Trem2*^R47H CSS^, and *Trem2* KO vs wild-type on control diet (FC=0.5; FDR<0.05). The numbers in the upper right and left corner of each volcano plot denote the number of genes displaying altered expression. d Venn diagram of DEGs between *Trem2*^R47H NSS^, *Trem2*^R47H CSS^, and *Trem2* KO vs wild-type on control diet. e Venn diagram of DEGs between CPZ treatment vs. control across 4 groups; wild-type, *Trem2*^R47H NSS^, *Trem2*^R47H CSS^, and *Trem2* KO. f Heatmap generated from DEGs of *Trem2*^R47H CSS ^(FDR<0.05 for CPZ vs. control) compared across mouse models highlighting *Trem2* expression across groups (FDR: CPZ vs control - WT= 9.22E-08; *Trem2*^R47H CSS^= 1.62E-06; *Trem2*^R47H NSS^= 0.008; *Trem2* KO = 0.018).

**Additional file 5:** **Supplemental Figure 4. **Comparison plot of CPZ PyWGCNA modules to published human AMP-AD modules indicating significant correlation between three major modules from CPZ and three different groups of AMP-AD modules.

**Additional file 6**: **Supplemental Figure 5. **Age and brain region–dependent sex differences in 5xFAD/*Trem2*^R47H^compared to 5xFAD. a-x Quantification of ThioS^+^ amyloid plaque number, volume, and mean intensity separated by sex (pink circles female, blue circles male) for (a-l) visual cortex and (m-x) subiculum at 4 and 12 months. n=5-6. Data are represented as mean ± SEM. Student’s t-test. Statistical significance is denoted by *p<0.05, **p<0.01, ***p<0.001, ****p<0.0001. Statistical trends are noted with the p value.

**Additional file 7**: **Supplemental Figure 6. **No change in cortical plaque intensity or OC^+^ volume in 5xFAD/*Trem2*^R47H^ compared to 5xFAD mice a, c Quantification of cortical Thio-S^+^ plaque density at (a) 4 and (c) 12 months. b, d Quantification of cortical OC^+^ volume at (b) 4 and (d) 12 months. n=8-12. Data are represented as mean ± SEM. Student’s t-test. Statistical significance is denoted by *p<0.05, **p<0.01, ***p<0.001, ****p<0.0001. Statistical trends are noted with the p value.

**Additional file 8**: **Supplemental Figure 7. **Microglia morphology analysis model. Representative Imaris v9.7 filament analysis screenshots of cortical IBA1^+^ cell morphology of 4 months WT, *Trem2*^R47H^, 5xFAD, and 5xFAD/*Trem2*^R47H^. Quantification shown in Figure 5 a,b.

**Additional file 9:** **Supplemental Figure 8. ***Trem2*^R47H^ induces sex-specific differences in microglia numbers at 4 months. a-d Number of IBA1^+^microglia in the (a-b) cortex and (c-d) subiculum at 4 months, separated by sex. e-f Quantification of percent colocalized volume of Thio-S^+^ and IBA1^+^ cell normalized to total Thio-S volume per field of view in the subiculum at 4 months separated by sex. n=5-6. Data are represented as mean ± SEM. Student’s t-test. Two-way ANOVA followed by Tukey’s post hoc tests to examine biologically relevant interactions. Statistical significance is denoted by *p<0.05, **p<0.01, ***p<0.001, ****p<0.0001.

**Additional file 10:** **Supplemental Figure 9. **Decreased astrocyte cell volume in 5xFAD/*Trem2*^R47H ^at 12-month. a, b Representative confocal images of visual cortex stained for dense-core plaques (Thio-S, green), immunolabeled reactive astrocytes (GFAP, red and S100β, blue). c, d At 4-months, quantification of GFAP^+^ cell density and average cell volume revealed no difference in cell number but larger cells in 5xFAD and 5xFAD/*Trem2*^R47H^ and their age-matched controls in subiculum. Cell number in the visual cortex is higher in 5xFAD and 5xFAD/*Trem2*^R47H^ compared to their age-matched controls despite no change in cell volume.  e, f Quantification of S100β^+^ cell density and average cell volume revealed increase in cell number of 5xFAD compared to WT and larger cells in 5xFAD compared to WT in the subiculum. In the cortex, there is no difference in S100β^+^ cell number but smaller cell volume in 5xFAD and 5xFAD/ *Trem2*^R47H^compared to their controls. g, h At 12-months, GFAP^+^ cell number in the subiculum of *Trem2*^R47H^, 5xFAD, and 5xFAD/ *Trem2*^R47H^mice were reduced compared to WT while cell volume is larger in 5xFAD compared to 5xFAD/ *Trem2*^R47H^. In the cortex, there are more GFAP^+^ cells in 5xFAD and 5xFAD/ *Trem2*^R47H^ compared to controls but no difference in size. i, j Quantification of S100β^+^ cell density and average cell volume revealed increases of both in 5xFAD and 5xFAD/*Trem2*^R47H^ compared to controls in the subiculum. In the cortex, there is no difference in S100β^+^ cell density but an increase in cell volume in 5xFAD and 5xFAD/ *Trem2*^R47H^. n=10-12. Data are represented as mean ± SEM. Two-way ANOVA followed by Tukey’s post hoc tests to examine biologically relevant interactions. Statistical significance is denoted by *p<0.05, **p<0.01, ***p<0.001, ****p<0.0001.

**Additional file 11:** **Supplemental Figure 10. ***Trem2*^R47H^ affects dystrophic neurites and axonal damage in an age- and sex-specific manner. a-d Sex-separate quantification of LAMP1^+^ volume normalized to plaque volume at (a,b) 4 months and (c,d) 12 months. e-j Measurement of NfL in (e,f) plasma, (g,h) soluble and (i,j) insoluble brain fraction at 4 and 12 months separated by sex. k-n Quantification of (k,l) number and (m-n) total NfL^+^ spheroid volume in the subiculum at 4 and 12 months compared between genotypes for each sex. n=5-6. Data are represented as mean ± SEM. Student’s t-test. Two-way ANOVA followed by Tukey’s post hoc tests to examine biologically relevant interactions. Statistical significance is denoted by *p<0.05, **p<0.01, ***p<0.001, ****p<0.0001.

**Additional file 12:** **Supplemental Figure 11. **Synaptic activity and immunostain. a-c Input-outputcurves measuring the magnitude of the (a) fEPSP slope and (b) fiber volley across a range of stimulation currents (10-100 µA) were comparable in fEPSP slope between 4 mo 5xFAD, *Trem2*^R47H^, and WT, but not 4 mo 5xFAD/*Trem2*^R47H^ (p<0.01). All three experimental groups showed a significant decrease in fiber volley amplitude with respect to WT controls (p <0.0001). c *Left,* Input-output curve comparing the amplitude of the fiber volley to the slope of the fEPSP response across a range of stimulation currents. *Right*, the mean slope of the input-output curves for each slice/group was significantly enhanced in slices from *Trem2*^R47H^ mice relative to WT controls. d Representative super-resolution images at 63X objective of CA1 from 4-month-old WT, *Trem2*^R47H^, 5xFAD, and 5xFAD/*Trem2*^R47H^ mice immunolabeled with Bassoon (BSN) for presynaptic elements (red; top panel) and PSD-95 for postsynaptic elements (green; bottom panel). e-f Quantification of (e) Bassoon^+^ and (f) PSD-95^+^ spots per µm^3^ showed a decrease in both pre- and post-synaptic puncta in 5xFAD and a trending rescue of presynaptic elements in 5xFAD/*Trem2*^R47H^ mice. g-i Input-output curves measuring the magnitude of the (g) fEPSP slope and (h) fiber volley across a range of stimulation currents (10-100 µA). (g)The fEPSP slopes generated in the latter part of the input-output curve were significantly reduced in slices from 12 mo 5xFAD, *Trem2*^R47H^, and 5xFAD/*Trem2*^R47H^ mice relative to WT controls (p<0.0001). (h) No measurable difference in fiber volley amplitude was found at any given stimulus between groups. i *Left,* Input-output curve comparing the amplitude of the fiber volley to the slope of the fEPSP response across a range of stimulation currents. *Right*, the mean slope of the input-output curves for each experimental slice/group was not significantly different from WT controls. j Representative super-resolution images at 63X objective of CA1 from 12-month-old WT, *Trem2*^R47H^, 5xFAD, and 5xFAD/*Trem2*^R47H^ mice immunolabeled with Bassoon for presynaptic elements (red; top panel) and PSD-95 for postsynaptic elements (green; bottom panel). k Bassoon^+^ pre-synaptic element decreases in 5xFAD mice compared to WT. l PSD-95^+^ spots per µm^3^ reveals decrease in post-synaptic puncta in *Trem2*^R47H^compared to WT mice and an increase in 5xFAD/*Trem2*^R47H ^compared to 5xFAD mice. n=10-12. Data are represented as mean ± SEM. Two-way ANOVA followed by Tukey’s post hoc tests to examine biologically relevant interactions. Statistical significance is denoted by *p<0.05, **p<0.01, ***p<0.001, ****p<0.0001.

**Additional file 13:** **Supplemental Figure 12. **a PCA was applied to the 5xFAD/*Trem2* dataset, replicating the separation of age in the first principal component. b-c Heatmaps generated from (b) *Trem2*-dependent and (c) *Trem2*-independent upregulated inflammatory genes in response to the CPZ challenge in Fig. 2 of WT, *Trem2*^R47H^, 5xFAD and 5xFAD/*Trem2*^R47H^at 4- and 12-month timepoints. d Module Trait relationship heatmap by PyWGCNA on WT, *Trem2*^R47H^, 5xFAD and 5xFAD/*Trem2*^R47H^. Color corresponding to correlation (red denotes positive correlation; blue denotes negative correlation) and the number in parenthesis shows relative significance of each correlation. The Darkgrey module was chosen as it is the inflammatory module and based on its significant correlation with the disease state. Light grey and Snow modules were chosen based on their significant correlation with the AMP-AD modules highlighted in Supplemental Fig. 13.

**Additional file 14:** **Supplemental Figure 13. **Overlapping WGCNA modules between 5xFAD/*Trem2*^R47H^and human AMP-AD. a Comparison plot of 5xFAD/*Trem2*^R47H^ WGCNA modules to published human AMP-AD modules confirming significant overlap between immune response cytokine signaling AMP-AD modules and Darkgrey module (highlighted in Fig.8); Lightgrey module with neuronal system modules, as well as Snow module with myelination. b Module eigengenes of Lightgrey plotted as bar graphs with WT, *Trem2*^R47H^, 5xFAD and 5xFAD/*Trem2*^R47H^at 4- and 12-month timepoints. Two data points were identified as outliers and removed. c Gene ontology of the Lightgrey module. d Module eigengenes of Snow plotted as bar graphs with WT, *Trem2*^R47H^, 5xFAD and 5xFAD/*Trem2*^R47H^at 4- and 12-month timepoints. e Gene ontology of the Snow module. Eigengene bar plots - n=10-15. Data are represented as mean ± SEM. Two-way ANOVA followed by Tukey’s post hoc tests to examine biologically relevant interactions. Statistical significance is denoted by *p<0.05, **p<0.01, ***p<0.001, ****p<0.0001.

**Additional file 15**: **Supplemental Figure 14. ***Trem2*^R47H^ alters expression of *Itgax and Cd74 *in an age-dependent mannera, b Representative confocal images of hippocampal subiculum in wild-type, *Trem2*^R47H^, 5xFAD, and 5xFAD/*Trem2*^R47H^ mice at (a) 4- and (b) 12-months old, stained with Amylo-Glo for dense-core plaques (green), immunolabeled with IBA1 for microglia (blue), CD11c (white), and CD74 (red) confirms reduced expression of *Itgax* (CD11c*) *and CD74. c, d 4-month TPM values of *Itgax* and *Cd74* plotted as bar graphs showed decreased expression between 5xFAD and 5xFAD/*Trem2*^R47H^ in both (c) *Itgax* and (d) *Cd74.* e, f Quantification of colocalization of IBA1 and (e) CD11c and (f) CD74 normalized to total IBA1 volume confirmed reduction in the respective gene expression. g, h 12-month-old TPM values of *Itgax* and *Cd74* from bulk cortical RNA sequencing data revealed a decrease in (g) *Itgax* but not (h) *Cd74*. i, j Quantification of 12-month colocalization of IBA1 and (i) CD11c and (j) CD74 normalized to total IBA1 volume confirmed changes in the respective gene expression. n=10-12. Data are represented as mean ± SEM. Two-way ANOVA followed by Tukey’s post hoc tests to examine biologically relevant interactions. Statistical significance is denoted by *p<0.05, **p<0.01, ***p<0.001, ****p<0.0001.

**Additional file 16**: **Supplemental Figure 15. **CRISPR-generated *Trem2*^R47H^ alleles and their impact on induction of cryptic splicing events.  DNA sequence alignment of part of exon 2 of mouse (*Mm*) *Trem2* from wild-type and four CRISPR-generated R47H encoding alleles (from top to bottom, Cheng-Hathaway *et. al* [34], *Trem2*^R47H CSS^(JAX 027918), Xiang *et. a*l [35] and *Trem2*^R47H NSS^ (JAX 034036, this study). The R47 coding triplet and amino acid in mouse is shown in green, with the G to A transition that changes the R to H in each CRISPR allele shown in red. Blue nucleotides in each CRISPR-generated allele denote synonymous DNA bases co-introduced with the G to A transition. The equivalent region of human (*Hs*) *TREM2* is shown at the top with nucleotide differences between mouse and human in brown. A summary of the presence of cryptic splicing and quantity of transcripts relative to the wild type allele is shown at the right of each CRISPR-generated allele (data from this study and [34, 35]. Evidence for cryptic splicing from the R47H *Trem2* allele described in Cheng-Hathaway *et. al* [34] is from transfection of cells with recombinant DNA expression constructs as reported [35]. The underlined AG dinucleotide denotes the end of the “intronic” sequence removed in events using the cryptic splice site (identified in Xiang *et. al* [35] and this study) with the corresponding underlined TGA stop codon produced by the cryptic splice event. The bottom of the figure shows a consensus sequence for a splice branch point (YUVAY; Y = T or C; V = A, C or G [73]) and a polypyrimidine tract, with bases matching the wildtype mouse *Trem2* sequence in green, which may stimulate cryptic splicing. Splice branch sequences in mammals are highly degenerate [74, 75]. Comparison of the sequence of each CRISPR-generated mouse R47H allele combined with results from studies using a cell culture-based assay, including analysis of the related human DNA sequence [35], does not provide clear insight into why transcripts from some mouse CRISPR-generated R47H alleles undergo cryptic splicing while those from the *Trem2* ^R47H NSS ^allele apparently do not.

**Additional file 17**: Supplemental Table 1. Primers for PCR amplification and sequencing for off-target analysis.  Forward (For) and reverse (Rev) primer sequences are listed for each potential off-target site. The off-target code corresponds to the panels in Supplemental Fig.2.

**Additional file 18:** **Supplemental Table 2. **Location of potential off-target sites for crRNA TMF1342 on mouse chromosome 17. The sequence of each of the 11 potential off-target sites on mouse chromosome 17 is listed (GRCm38/mm10 nucleotide numbering), as well as the intended target site within *Trem2*. Mismatches to the crRNA guide are indicated by lowercase bold letters. Blue text denotes the 8-nucleotide seed region proximal to the PAM site. The vertical line at the 3’ end of each target or off-target site indicates the boundary between the gRNA target and the adjacent Cas9 PAM (NGG) sequence. The column labeled NSS vs WT p value lists the p-value for gene expression difference for each of the 11 potential off-target genes in whole brain between 15-week-old mice (n=8 for WT and n=4 for *Trem2*^R47H NSS^, all males, analyzed via bulk RNA-seq (edgeR, p-values stated).

## Data Availability

Protocols, data, and results are available via the AD Knowledge Portal (https://adknowledgeportal.synapse.org). The AD Knowledge Portal is a platform for accessing data, analyses, and tools generated by the Accelerating Medicines Partnership (AMP-AD) Target Discovery Program and other National Institute on Aging (NIA)-supported programs to enable open-science practices and accelerate translational learning. The data, analyses and tools are shared early in the research cycle without a publication embargo on secondary use. Data is available for general research use according to the following requirements for data access and data attribution (https://adknowledgeportal.org/DataAccess/Instructions). Data can be accessed in an interactive matter at UCI Mouse Mind Explorer (admodelexplorer.org). The *Trem2*^R47H NSS^ model is available from The Jackson Laboratory (Stock #034,036) without restrictions on its use by both academic and commercial users. The content is solely the responsibility of the authors and does not necessarily represent the official view of the National Institutes of Health.

## Abbreviations

AD: Alzheimer’s disease
TREM2: Triggering receptor expressed on myeloid cells 2
Aβ: Amyloid-beta
NSS: Normal splice Site
CSS: Cryptic splice Site
GWAS: Genome-wide association studies
LOAD: Late-onset alzheimer’s disease
PBS: Phosphate buffered saline
PFA: Paraformaldehyde
fEPSPs: Field excitatory postsynaptic potentials
PPF: Pair-pulse facilitation
LTP: Long-term potentiation
aCSF: Artificial cerebrospinal fluid
WGCNA: Weighted gene correlation network analysis
CPZ: Cuprizone
ThioS: Thiosflavin-S
DEGs: Differentially expressed genes
DAM: Disease-associated microglia
NfL: Neurofilament light chain
LTP: Long-term potentiation
APP: Amyloid precursor protein
PSEN: Presenilin
DAP12: DNAX-activation protein of 12 kb
SYK: Spleen tyrosine kinase
PI3K: Phosphatidylinoaitol-3-kinase
BAC: Bacterial artificial chromosome
ITAM: Immunoreceptor tyrosine-based activation motifs
WT: Wild-type
