# Supplementary figures and images for: A Trem2R47H mouse model without cryptic splicing drives age- and disease-dependent tissue damage and synaptic loss in response to plaques
Source: Mol Neurodegener. 2023 Feb 17;18:12. doi: 10.1186/s13024-023-00598-4 (PMC9938579; doi:10.1186/s13024-023-00598-4)

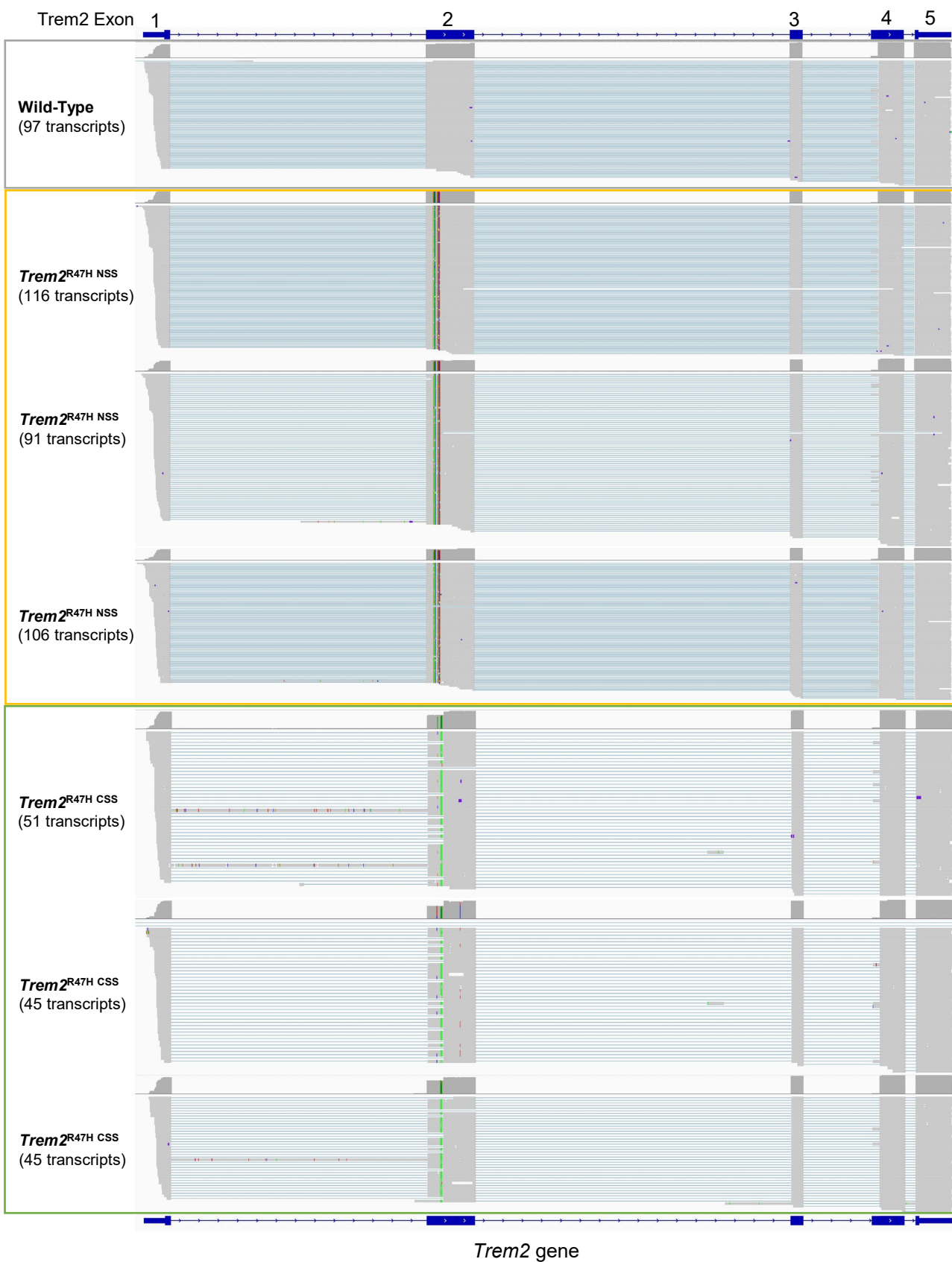

Supplement: Supplementary file 2 — Additional file 2: Supplemental Figure 1. Long-read RNA-seq analysis of Trem2 transcripts from whole brain of 15-wk old wild type, homozygous Trem2R47H NSS and homozygous Trem2R47H CSS mice. For each of the three genotypes, independent transcripts are shown in grey, aligned to the known five exons of Trem2 (dark blue) with inferred intronic sequences in light blue. Total transcripts from one wild-type mouse are shown, and three from both Trem2R47H NSS and Trem2R47H CSS brains. The top part of each figure shows a compressed view of the transcripts. For Trem2R47H NSS and Trem2R47H CSS, the colored regions within exon 2 denote DNA bases that vary from the wildtype reference sequence. As reported [35], the Trem2R47H CSS allele produces a significant number of transcripts that use a cryptic splice site within exon 2, which are not observed in either the wild type or Trem2R47H NSS samples. [file 13024_2023_598_MOESM2_ESM.pdf]

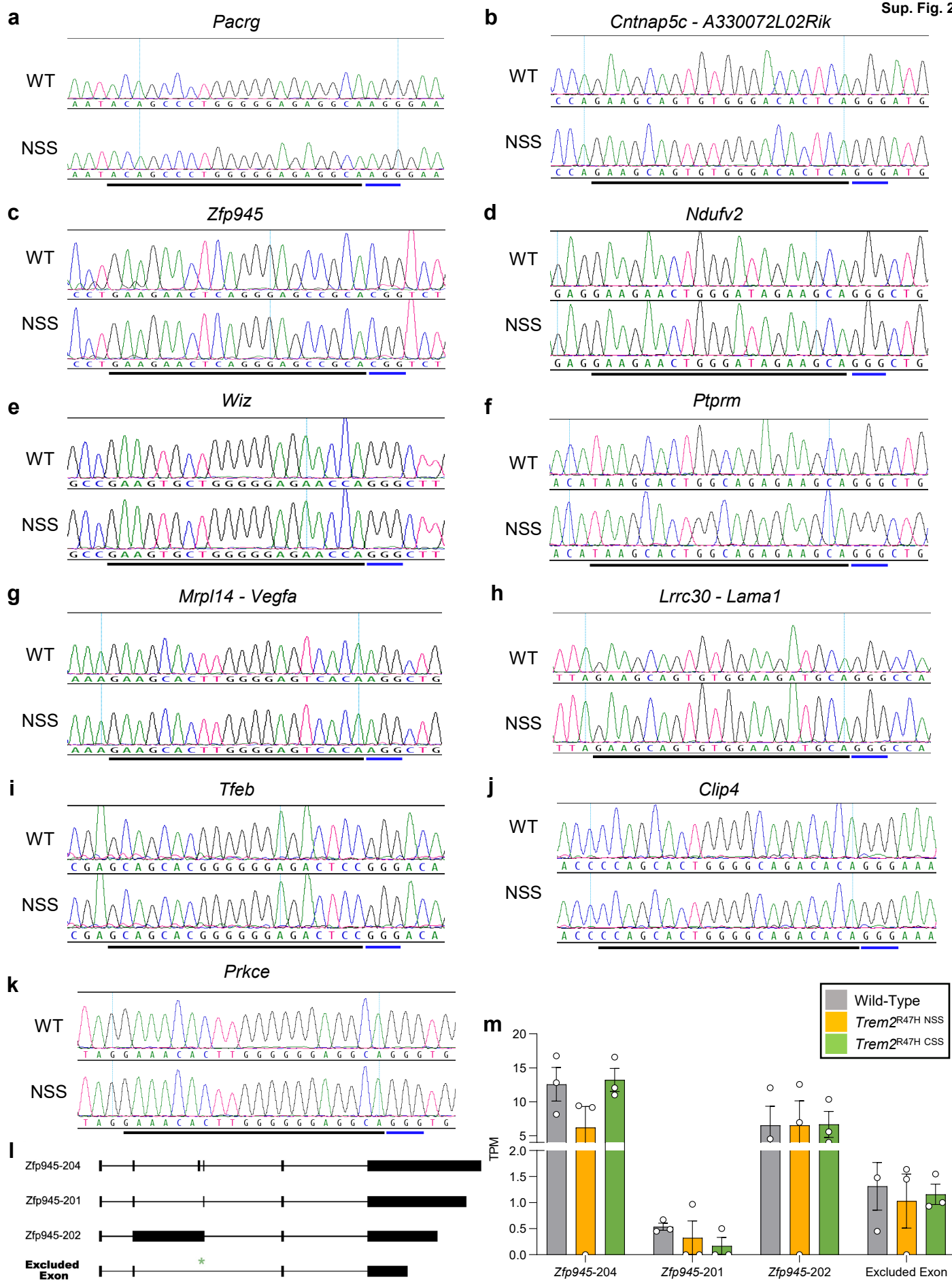

Supplement: Supplementary file 3 — Additional file 3: Supplemental Figure 2. Off-target site activity analysis for crRNA TMF1342 on mouse chromosome 17. a-k No difference was found in sequence between the C57BL/6J WT and Trem2R47H NSSalleles at each of the 11 potential off-target sites analyzed. The black underline denotes the crRNA target sequence while the blue underline denotes the NGG PAM site. l-m Long-read RNA-seq analysis of transcripts from one potential off-target locus (Zfp945) in brain shows no difference in the level of each isoform. The green asterisk denotes the excluded exon compared to Zfp945-201. [file 13024_2023_598_MOESM3_ESM.pdf]

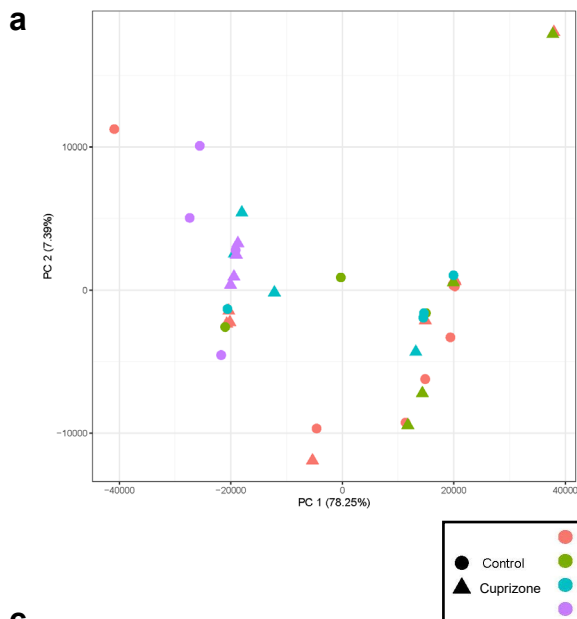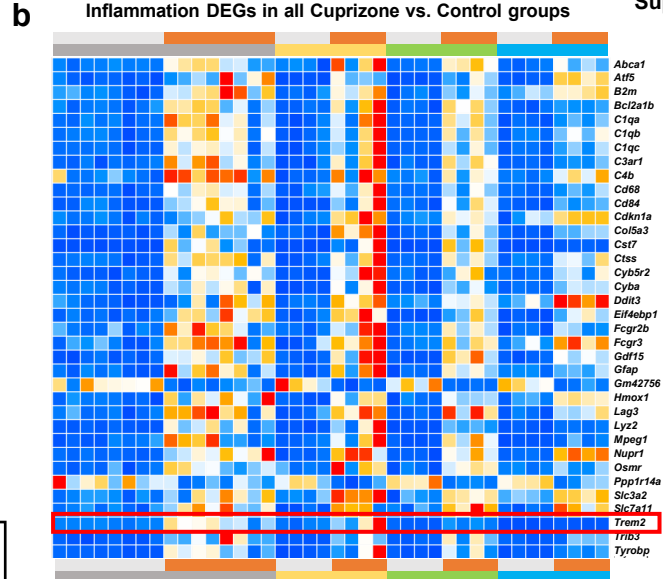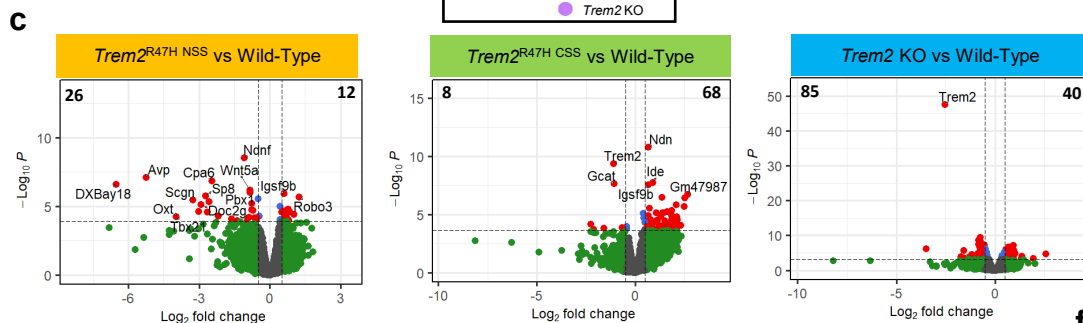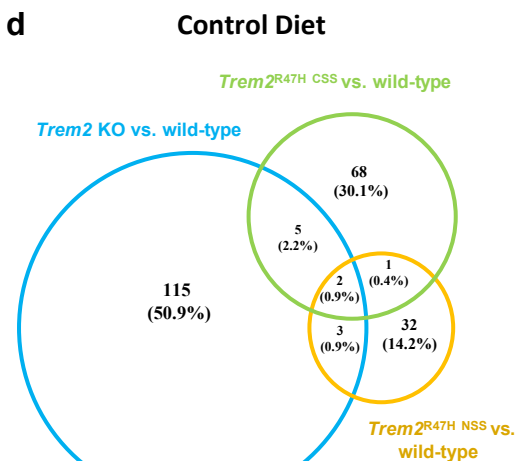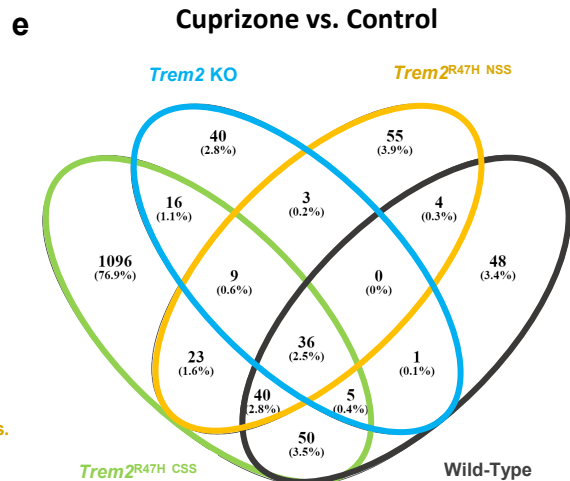

**f**

*Trem2*<sup>R47H</sup> CSS Cuprizone vs. Control DEGs

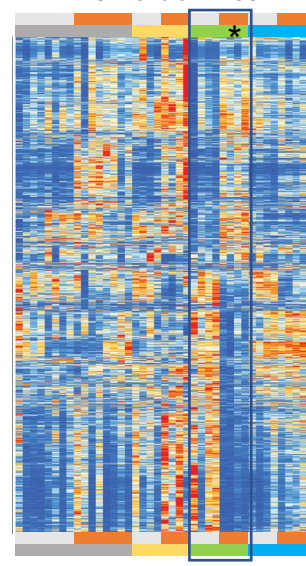

Supplement: Supplementary file 4 — Additional file 4: Supplemental Figure 3. Gene expression profile differences between Trem2R47H NSS, Trem2R47H CSSand Trem2 KO at steady-state and following cuprizone challenge. a PCA plot of cuprizone (CPZ) dataset. b Heatmap generated from selected Trem2-independent upregulated inflammatory DEGs from Fig. 2b and d. c Volcano plot of DEG, displaying fold change of genes (log2 scale) and P values (−log10 scale) between Trem2R47H NSS, Trem2R47H CSS, and Trem2 KO vs wild-type on control diet (FC=0.5; FDR<0.05). The numbers in the upper right and left corner of each volcano plot denote the number of genes displaying altered expression. d Venn diagram of DEGs between Trem2R47H NSS, Trem2R47H CSS, and Trem2 KO vs wild-type on control diet. e Venn diagram of DEGs between CPZ treatment vs. control across 4 groups; wild-type, Trem2R47H NSS, Trem2R47H CSS, and Trem2 KO. f Heatmap generated from DEGs of Trem2R47H CSS (FDR<0.05 for CPZ vs. control) compared across mouse models highlighting Trem2 expression across groups (FDR: CPZ vs control - WT= 9.22E-08; Trem2R47H CSS= 1.62E-06; Trem2R47H NSS= 0.008; Trem2 KO = 0.018). [file 13024_2023_598_MOESM4_ESM.pdf]

## Trem2 Cuprizone modules

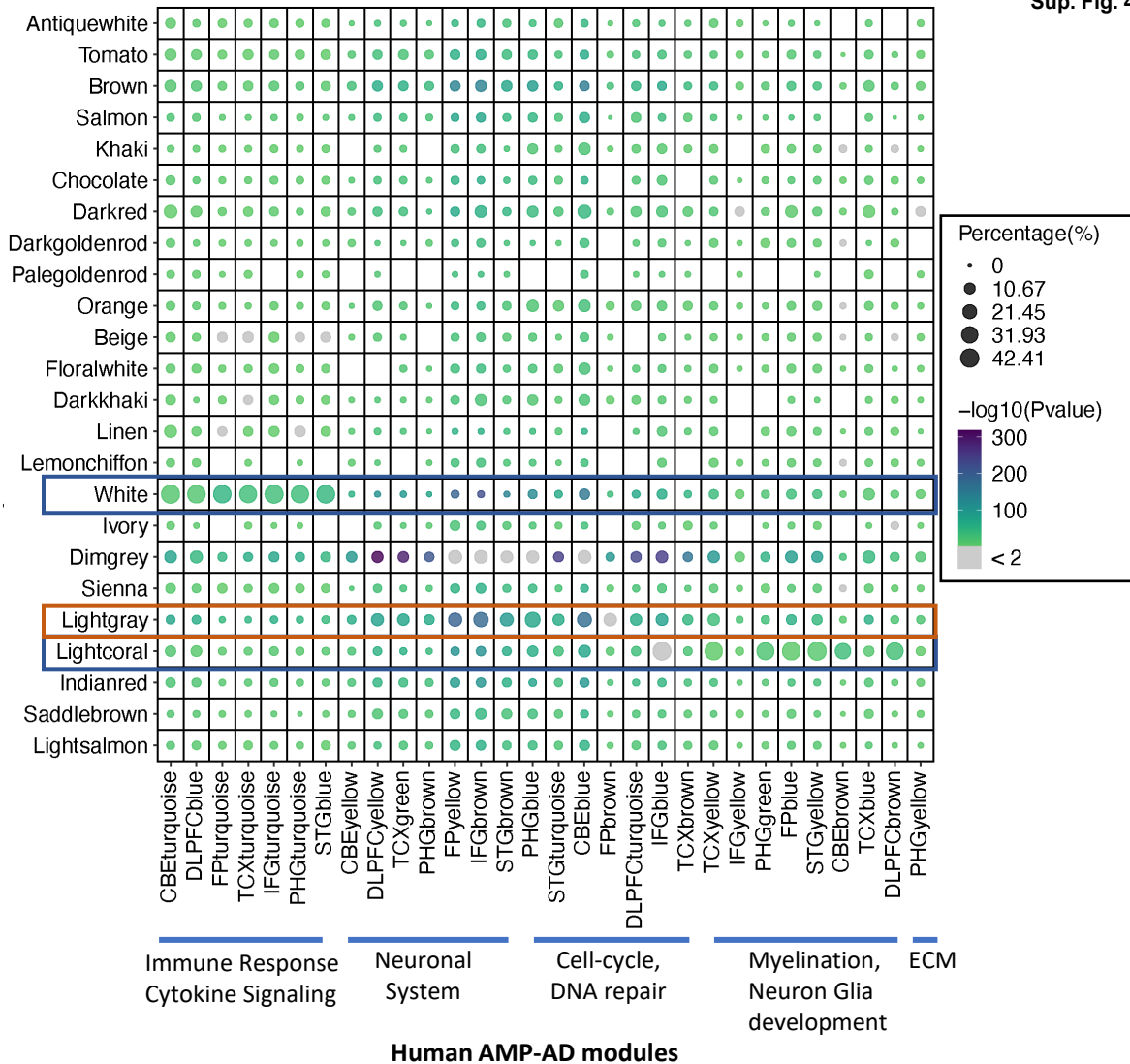

Supplement: Supplementary file 5 — Additional file 5: Supplemental Figure 4. Comparison plot of CPZ PyWGCNA modules to published human AMP-AD modules indicating significant correlation between three major modules from CPZ and three different groups of AMP-AD modules. [file 13024_2023_598_MOESM5_ESM.pdf]

4 month

12 month

## Visual Cortex

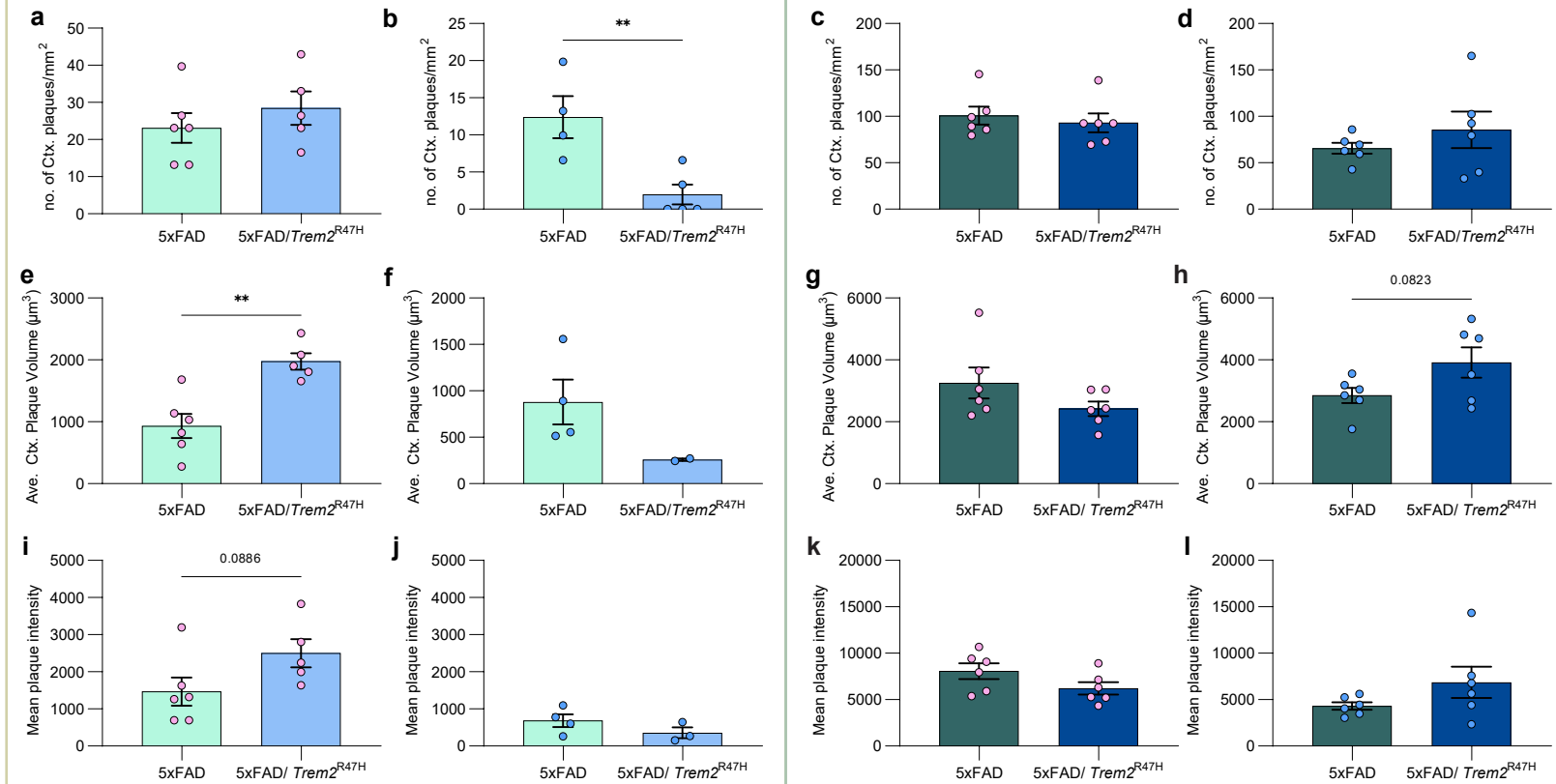

## Subiculum

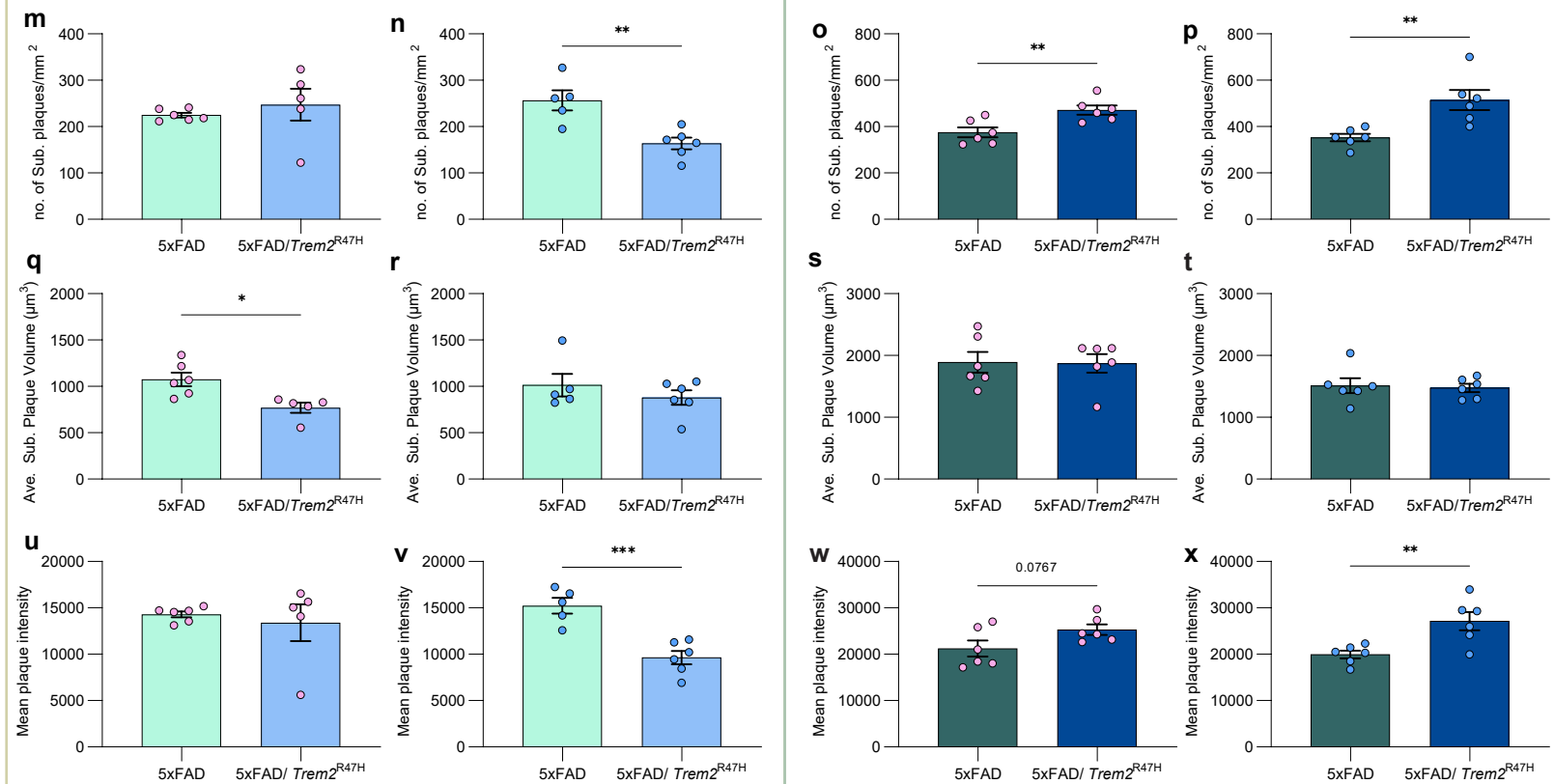

Supplement: Supplementary file 6 — Additional file 6: Supplemental Figure 5. Age and brain region–dependent sex differences in 5xFAD/Trem2R47Hcompared to 5xFAD. a-x Quantification of ThioS+ amyloid plaque number, volume, and mean intensity separated by sex (pink circles female, blue circles male) for (a-l) visual cortex and (m-x) subiculum at 4 and 12 months. n=5-6. Data are represented as mean ± SEM. Student’s t-test. Statistical significance is denoted by *p<0.05, **p<0.01, ***p<0.001, ****p<0.0001. Statistical trends are noted with the p value. [file 13024_2023_598_MOESM6_ESM.pdf]

4 month

Visual cortex

**a**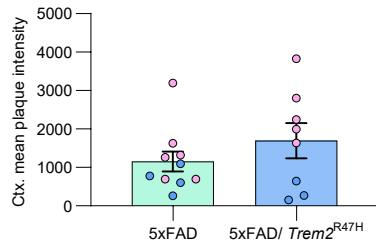**b**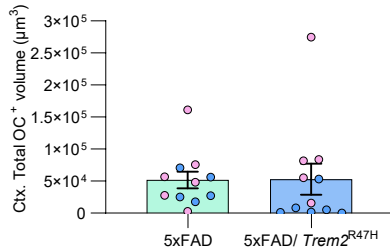

12 month

Visual cortex

**c**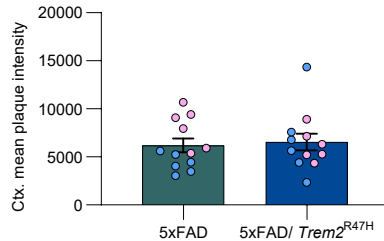**d**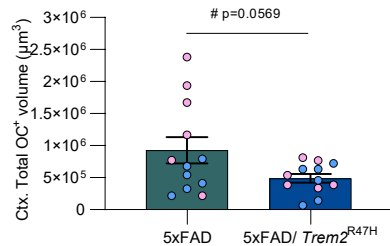

Supplement: Supplementary file 7 — Additional file 7: Supplemental Figure 6. No change in cortical plaque intensity or OC+ volume in 5xFAD/Trem2R47H compared to 5xFAD mice a, c Quantification of cortical Thio-S+ plaque density at (a) 4 and (c) 12 months. b, d Quantification of cortical OC+ volume at (b) 4 and (d) 12 months. n=8-12. Data are represented as mean ± SEM. Student’s t-test. Statistical significance is denoted by *p<0.05, **p<0.01, ***p<0.001, ****p<0.0001. Statistical trends are noted with the p value. [file 13024_2023_598_MOESM7_ESM.pdf]

WT

*Trem2*<sup>R47H</sup>

5xFAD

5xFAD/ *Trem2*<sup>R47H</sup>

Visual Cortex

15  $\mu$ m

IBA1

15  $\mu$ m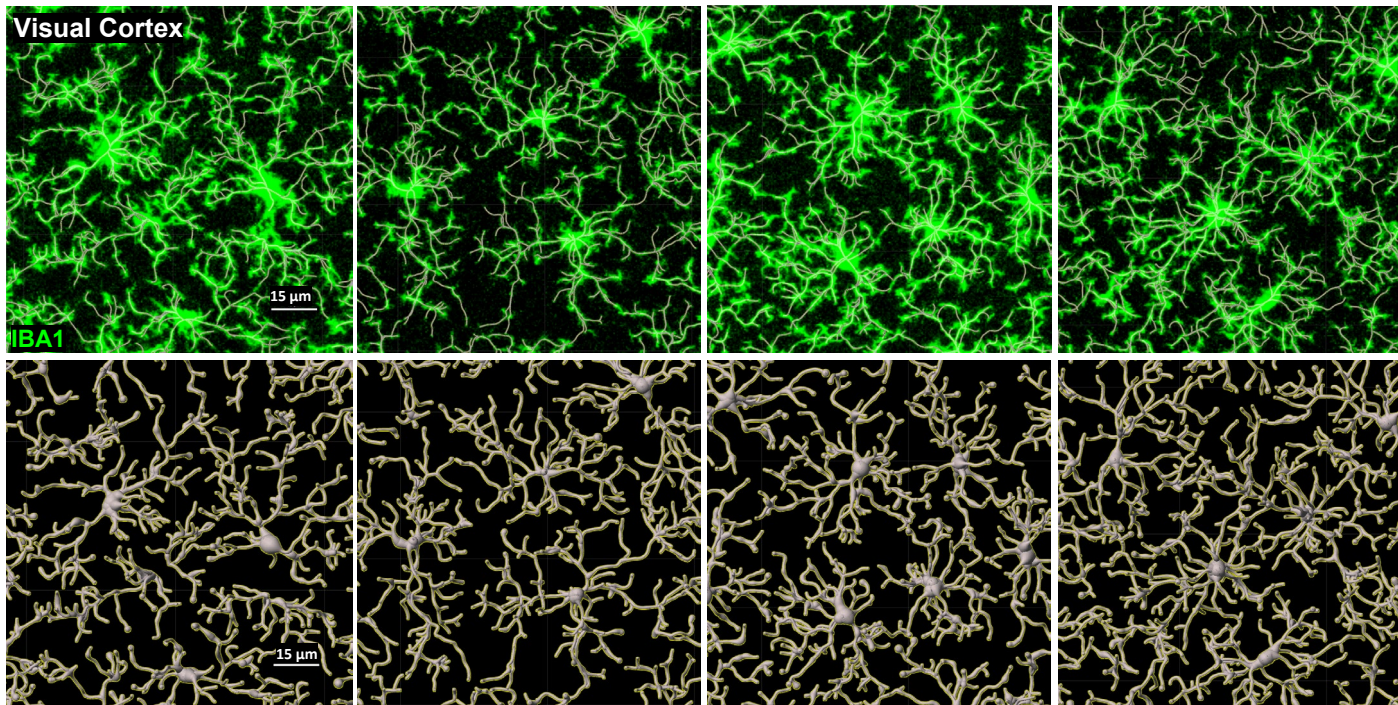

Supplement: Supplementary file 8 — Additional file 8: Supplemental Figure 7. Microglia morphology analysis model. Representative Imaris v9.7 filament analysis screenshots of cortical IBA1+ cell morphology of 4 months WT, Trem2R47H, 5xFAD, and 5xFAD/Trem2R47H. Quantification shown in Figure 5 a,b. [file 13024_2023_598_MOESM8_ESM.pdf]

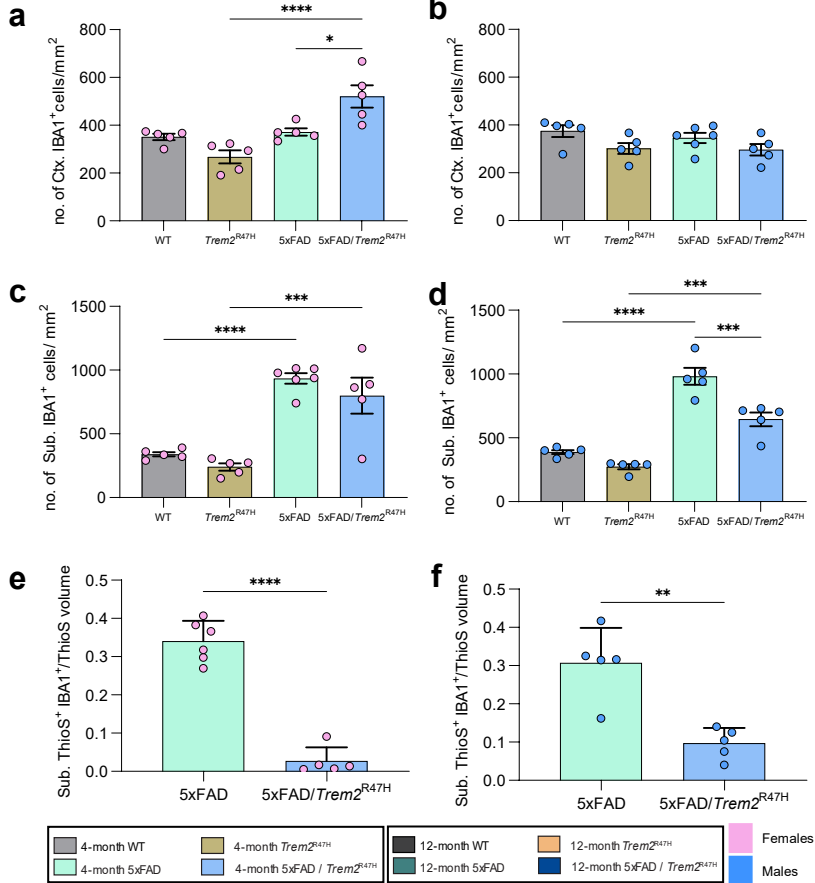

Supplement: Supplementary file 9 — Additional file 9: Supplemental Figure 8. Trem2R47H induces sex-specific differences in microglia numbers at 4 months. a-d Number of IBA1+microglia in the (a-b) cortex and (c-d) subiculum at 4 months, separated by sex. e-f Quantification of percent colocalized volume of Thio-S+ and IBA1+ cell normalized to total Thio-S volume per field of view in the subiculum at 4 months separated by sex. n=5-6. Data are represented as mean ± SEM. Student’s t-test. Two-way ANOVA followed by Tukey’s post hoc tests to examine biologically relevant interactions. Statistical significance is denoted by *p<0.05, **p<0.01, ***p<0.001, ****p<0.0001. [file 13024_2023_598_MOESM9_ESM.pdf]

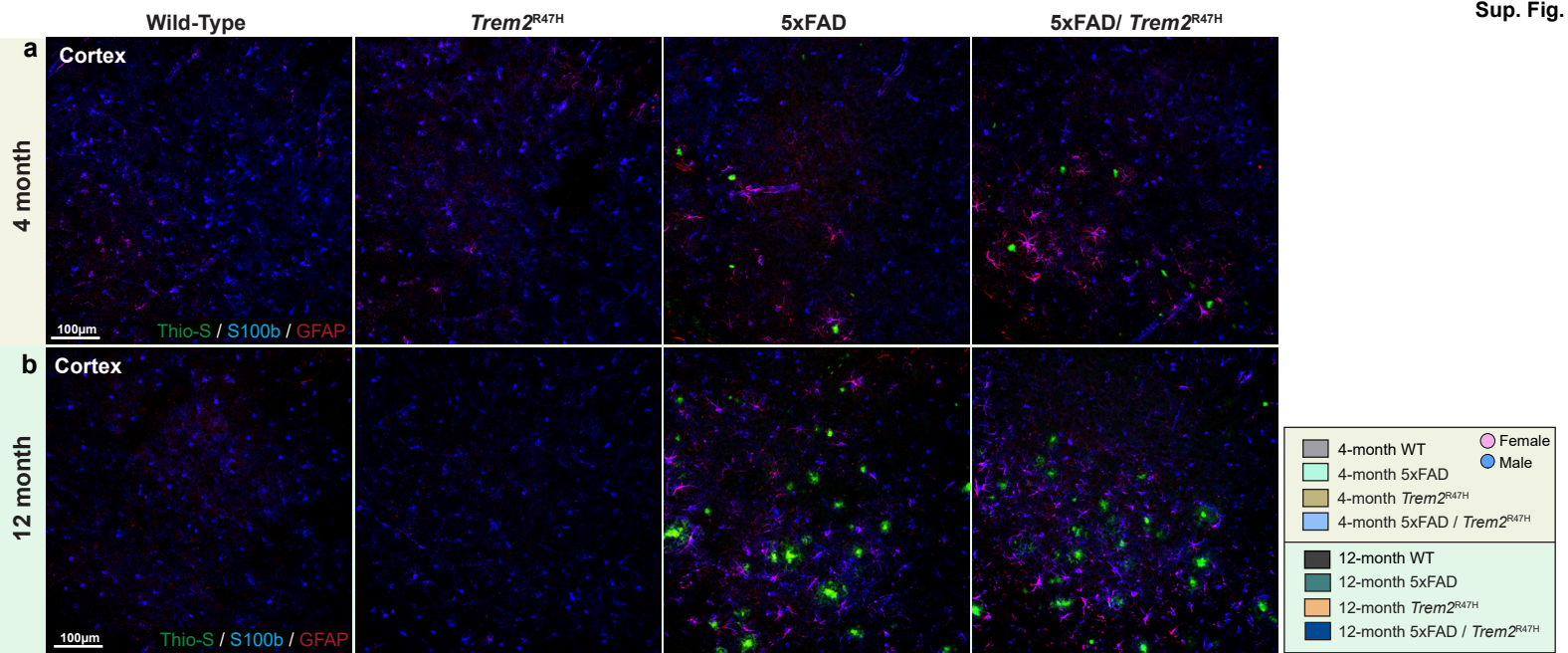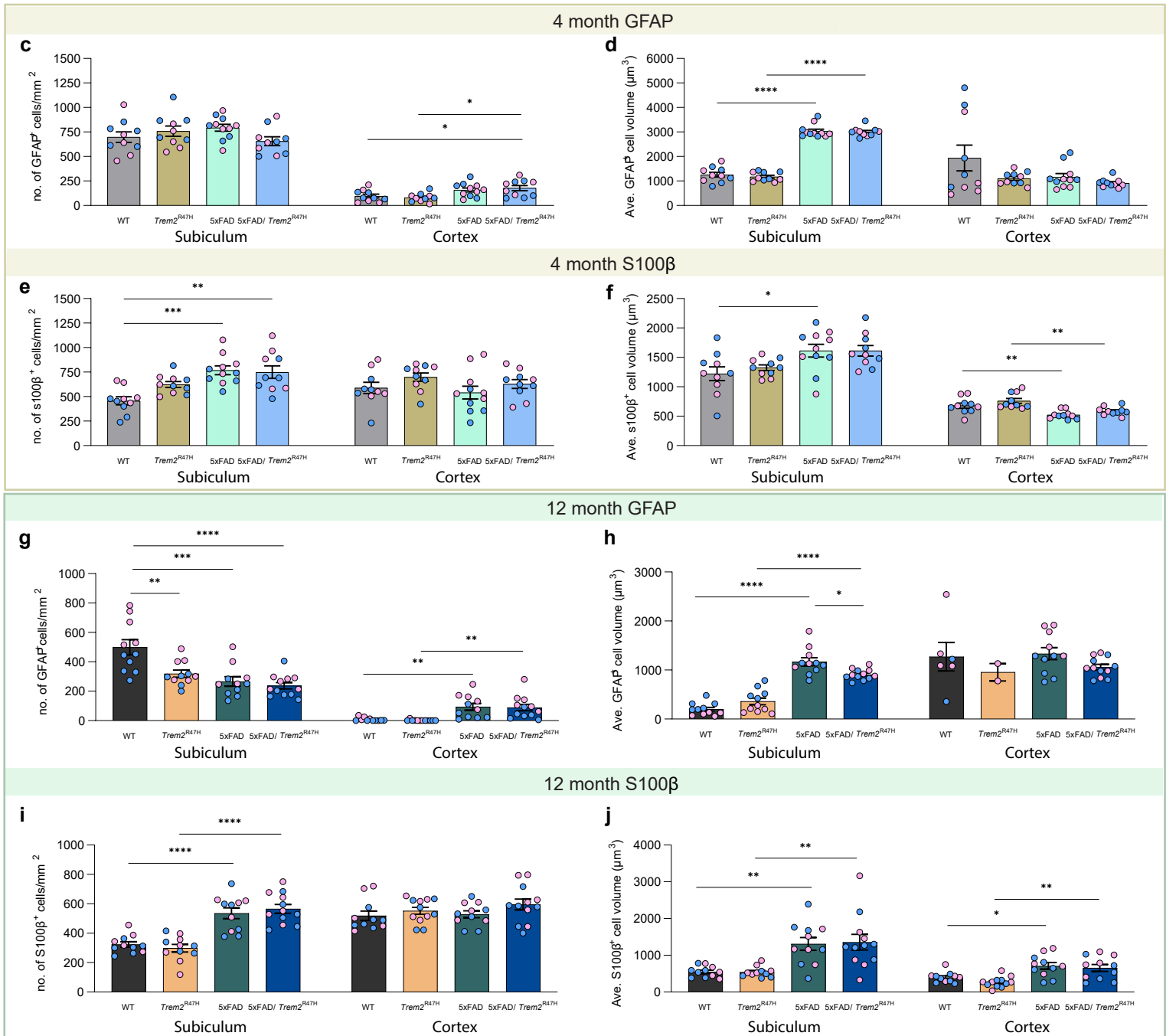

Supplement: Supplementary file 10 — Additional file 10: Supplemental Figure 9. Decreased astrocyte cell volume in 5xFAD/Trem2R47H at 12-month. a, b Representative confocal images of visual cortex stained for dense-core plaques (Thio-S, green), immunolabeled reactive astrocytes (GFAP, red and S100β, blue). c, d At 4-months, quantification of GFAP+ cell density and average cell volume revealed no difference in cell number but larger cells in 5xFAD and 5xFAD/Trem2R47H and their age-matched controls in subiculum. Cell number in the visual cortex is higher in 5xFAD and 5xFAD/Trem2R47H compared to their age-matched controls despite no change in cell volume. e, f Quantification of S100β+ cell density and average cell volume revealed increase in cell number of 5xFAD compared to WT and larger cells in 5xFAD compared to WT in the subiculum. In the cortex, there is no difference in S100β+ cell number but smaller cell volume in 5xFAD and 5xFAD/ Trem2R47Hcompared to their controls. g, h At 12-months, GFAP+ cell number in the subiculum of Trem2R47H, 5xFAD, and 5xFAD/ Trem2R47Hmice were reduced compared to WT while cell volume is larger in 5xFAD compared to 5xFAD/ Trem2R47H. In the cortex, there are more GFAP+ cells in 5xFAD and 5xFAD/ Trem2R47H compared to controls but no difference in size. i, j Quantification of S100β+ cell density and average cell volume revealed increases of both in 5xFAD and 5xFAD/Trem2R47H compared to controls in the subiculum. In the cortex, there is no difference in S100β+ cell density but an increase in cell volume in 5xFAD and 5xFAD/ Trem2R47H. n=10-12. Data are represented as mean ± SEM. Two-way ANOVA followed by Tukey’s post hoc tests to examine biologically relevant interactions. Statistical significance is denoted by *p<0.05, **p<0.01, ***p<0.001, ****p<0.0001. [file 13024_2023_598_MOESM10_ESM.pdf]

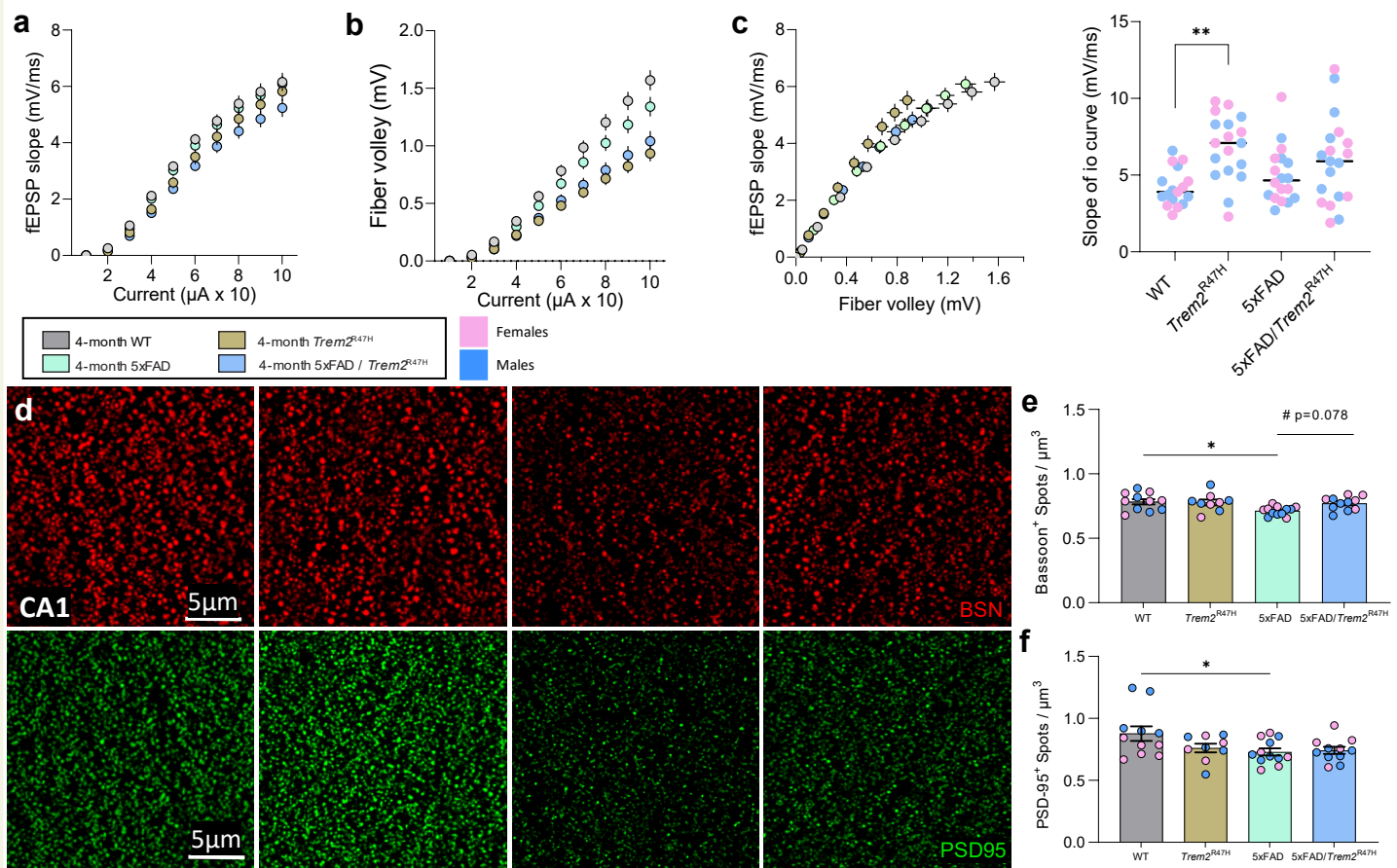

12 months

Supplement: Supplementary file 12 — Additional file 12: Supplemental Figure 11. Synaptic activity and immunostain. a-c Input-outputcurves measuring the magnitude of the (a) fEPSP slope and (b) fiber volley across a range of stimulation currents (10-100 µA) were comparable in fEPSP slope between 4 mo 5xFAD, Trem2R47H, and WT, but not 4 mo 5xFAD/Trem2R47H (p<0.01). All three experimental groups showed a significant decrease in fiber volley amplitude with respect to WT controls (p <0.0001). c Left, Input-output curve comparing the amplitude of the fiber volley to the slope of the fEPSP response across a range of stimulation currents. Right, the mean slope of the input-output curves for each slice/group was significantly enhanced in slices from Trem2R47H mice relative to WT controls. d Representative super-resolution images at 63X objective of CA1 from 4-month-old WT, Trem2R47H, 5xFAD, and 5xFAD/Trem2R47H mice immunolabeled with Bassoon (BSN) for presynaptic elements (red; top panel) and PSD-95 for postsynaptic elements (green; bottom panel). e-f Quantification of (e) Bassoon+ and (f) PSD-95+ spots per µm3 showed a decrease in both pre- and post-synaptic puncta in 5xFAD and a trending rescue of presynaptic elements in 5xFAD/Trem2R47H mice. g-i Input-output curves measuring the magnitude of the (g) fEPSP slope and (h) fiber volley across a range of stimulation currents (10-100 µA). (g)The fEPSP slopes generated in the latter part of the input-output curve were significantly reduced in slices from 12 mo 5xFAD, Trem2R47H, and 5xFAD/Trem2R47H mice relative to WT controls (p<0.0001). (h) No measurable difference in fiber volley amplitude was found at any given stimulus between groups. i Left, Input-output curve comparing the amplitude of the fiber volley to the slope of the fEPSP response across a range of stimulation currents. Right, the mean slope of the input-output curves for each experimental slice/group was not significantly different from WT controls. j Representative super-resolution images at 63X obje [file 13024_2023_598_MOESM12_ESM.pdf]

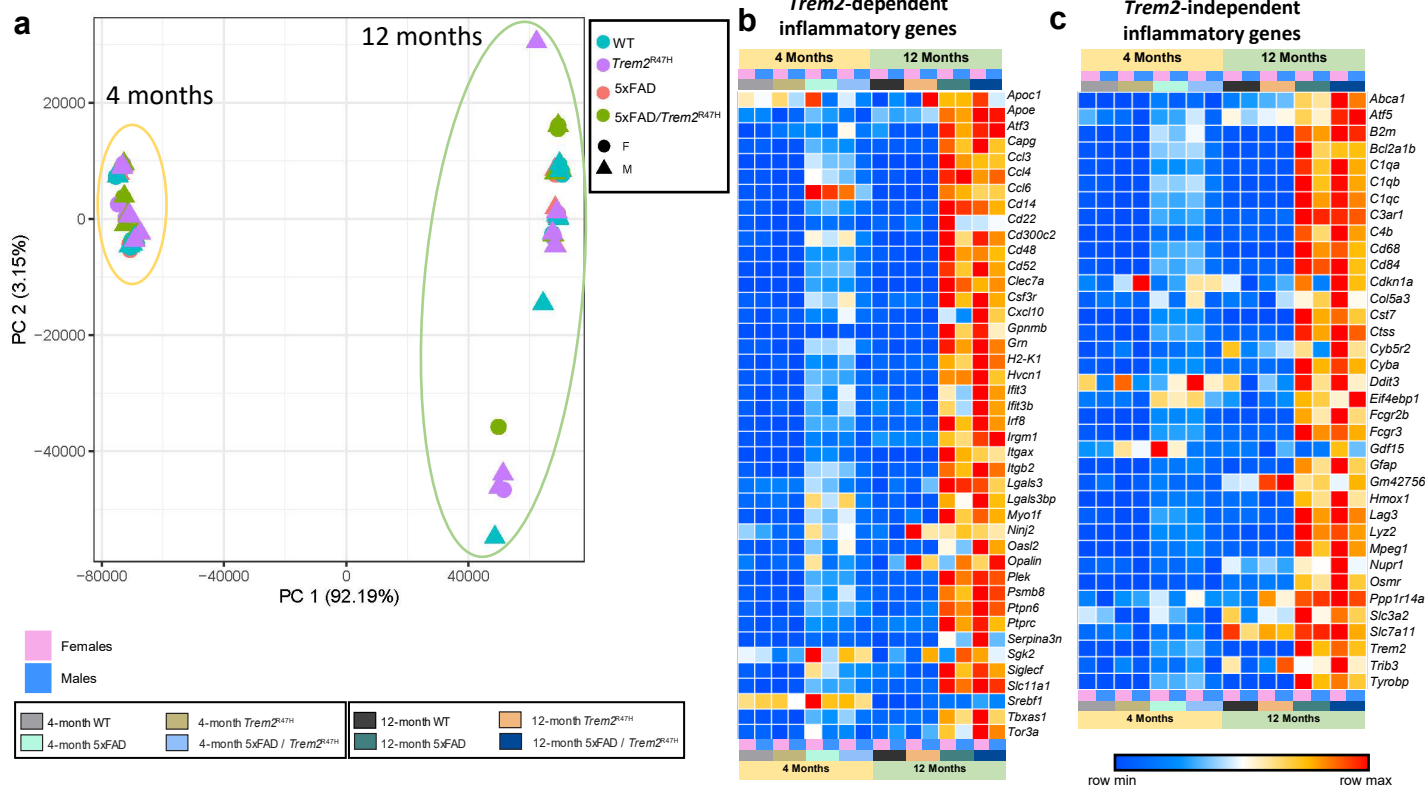

**d** WGCNA identified module – trait relationships

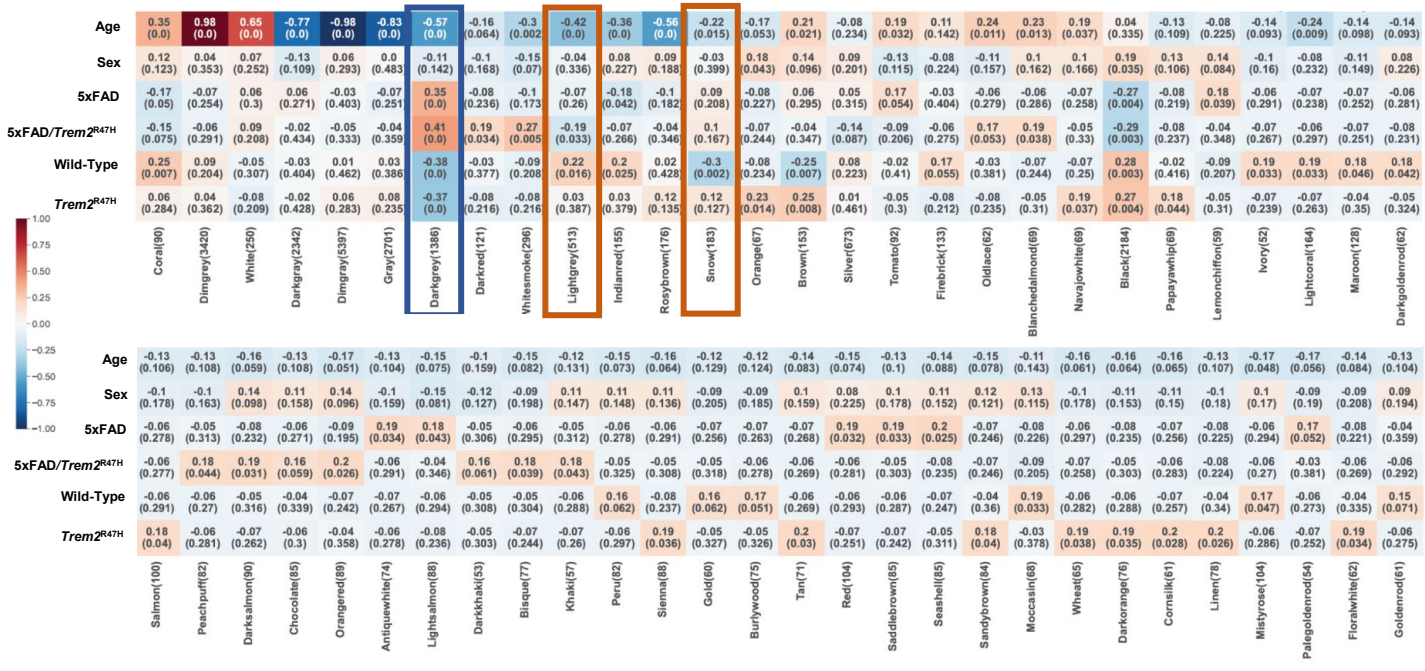

Supplement: Supplementary file 13 — Additional file 13: Supplemental Figure 12. a PCA was applied to the 5xFAD/Trem2 dataset, replicating the separation of age in the first principal component. b-c Heatmaps generated from (b) Trem2-dependent and (c) Trem2-independent upregulated inflammatory genes in response to the CPZ challenge in Fig. 2 of WT, Trem2R47H, 5xFAD and 5xFAD/Trem2R47Hat 4- and 12-month timepoints. d Module Trait relationship heatmap by PyWGCNA on WT, Trem2R47H, 5xFAD and 5xFAD/Trem2R47H. Color corresponding to correlation (red denotes positive correlation; blue denotes negative correlation) and the number in parenthesis shows relative significance of each correlation. The Darkgrey module was chosen as it is the inflammatory module and based on its significant correlation with the disease state. Light grey and Snow modules were chosen based on their significant correlation with the AMP-AD modules highlighted in Supplemental Fig. 13. [file 13024_2023_598_MOESM13_ESM.pdf]

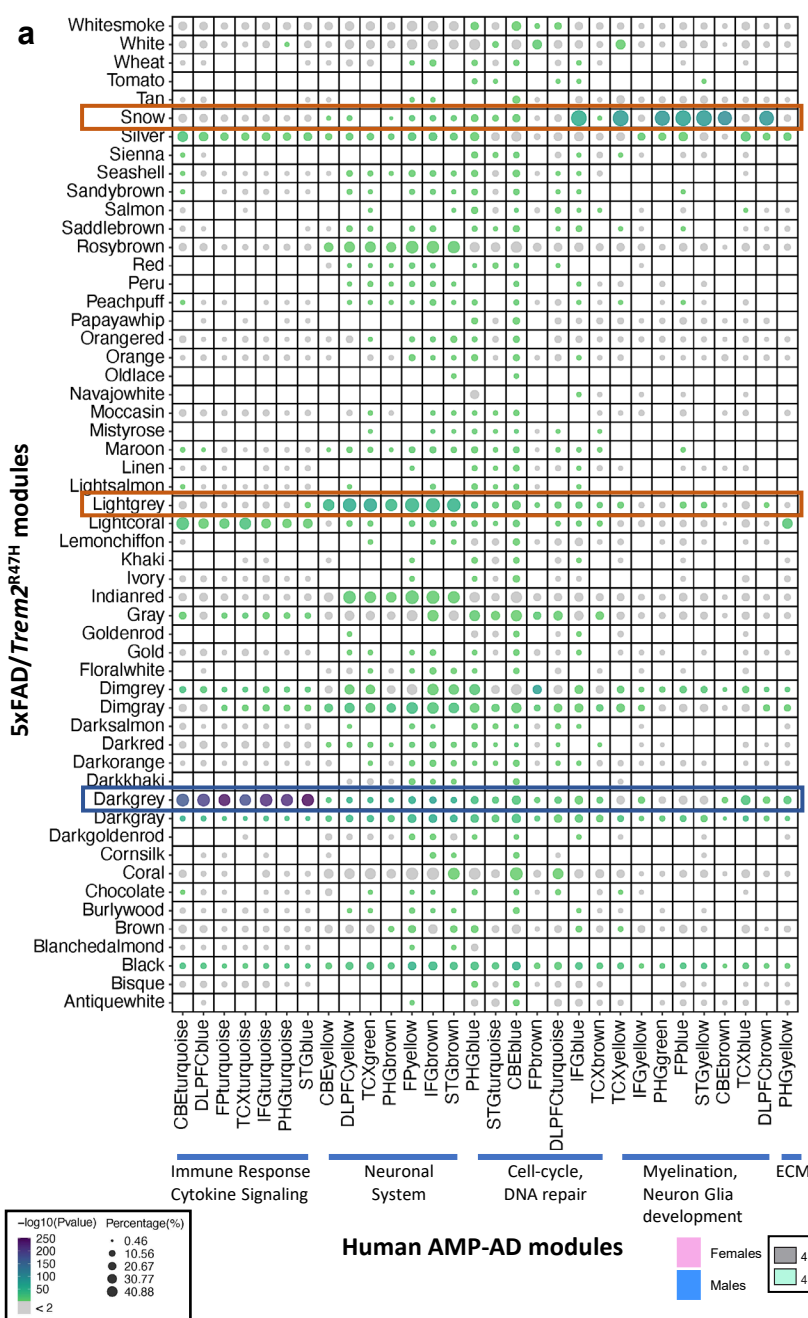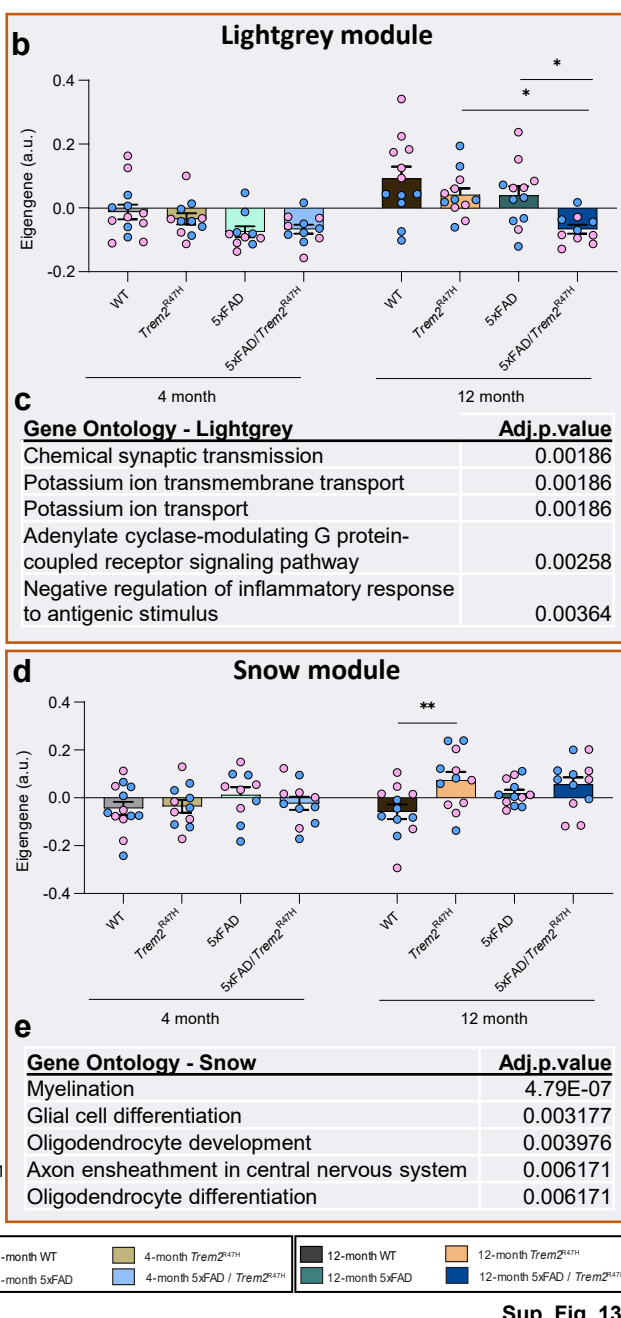

Supplement: Supplementary file 14 — Additional file 14: Supplemental Figure 13. Overlapping WGCNA modules between 5xFAD/Trem2R47Hand human AMP-AD. a Comparison plot of 5xFAD/Trem2R47H WGCNA modules to published human AMP-AD modules confirming significant overlap between immune response cytokine signaling AMP-AD modules and Darkgrey module (highlighted in Fig.8); Lightgrey module with neuronal system modules, as well as Snow module with myelination. b Module eigengenes of Lightgrey plotted as bar graphs with WT, Trem2R47H, 5xFAD and 5xFAD/Trem2R47Hat 4- and 12-month timepoints. Two data points were identified as outliers and removed. c Gene ontology of the Lightgrey module. d Module eigengenes of Snow plotted as bar graphs with WT, Trem2R47H, 5xFAD and 5xFAD/Trem2R47Hat 4- and 12-month timepoints. e Gene ontology of the Snow module. Eigengene bar plots - n=10-15. Data are represented as mean ± SEM. Two-way ANOVA followed by Tukey’s post hoc tests to examine biologically relevant interactions. Statistical significance is denoted by *p<0.05, **p<0.01, ***p<0.001, ****p<0.0001. [file 13024_2023_598_MOESM14_ESM.pdf]

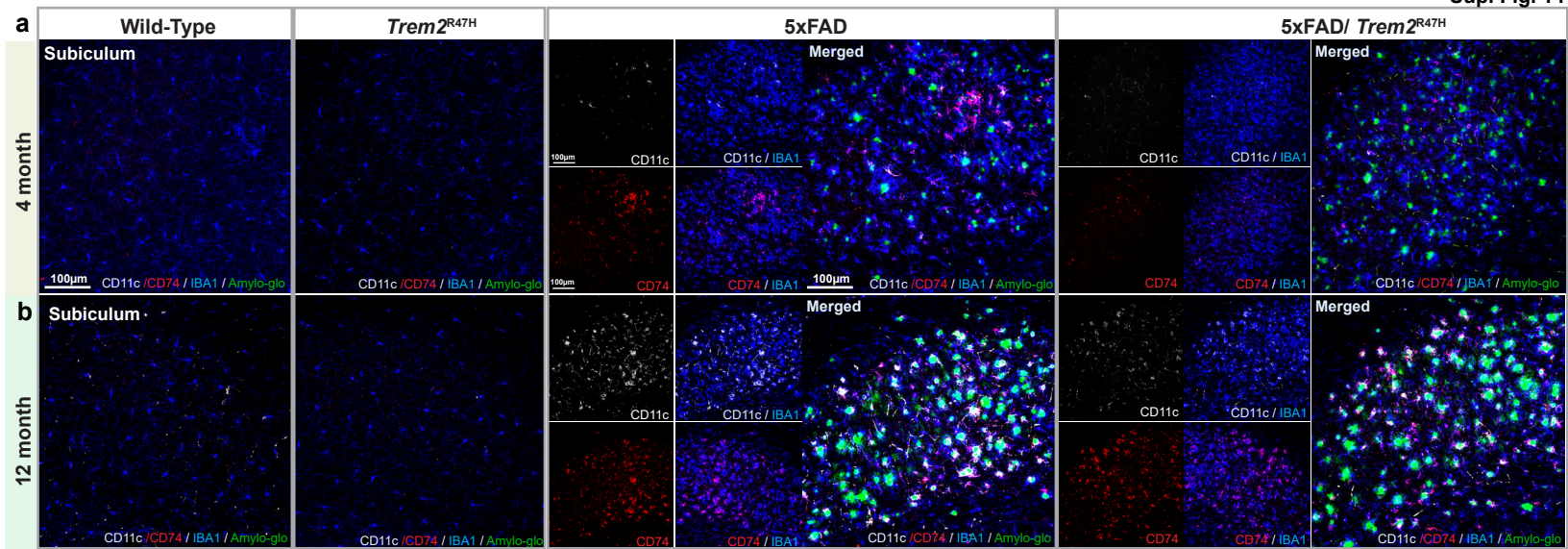

4 month

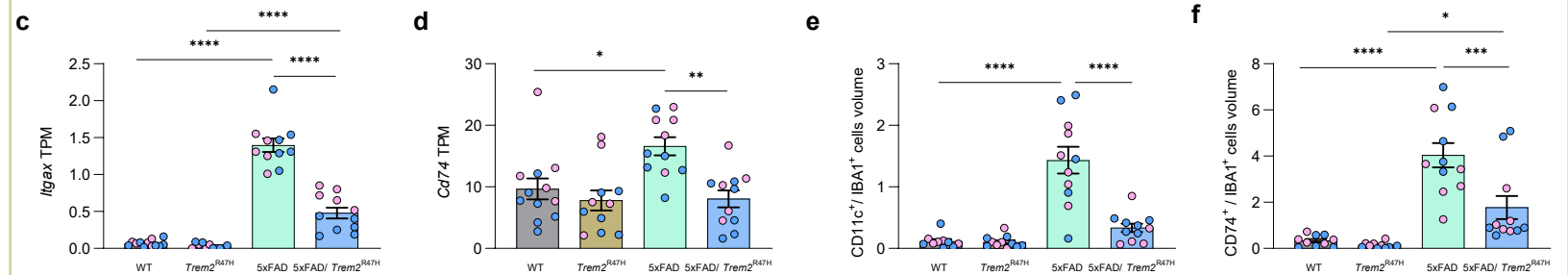

12 month

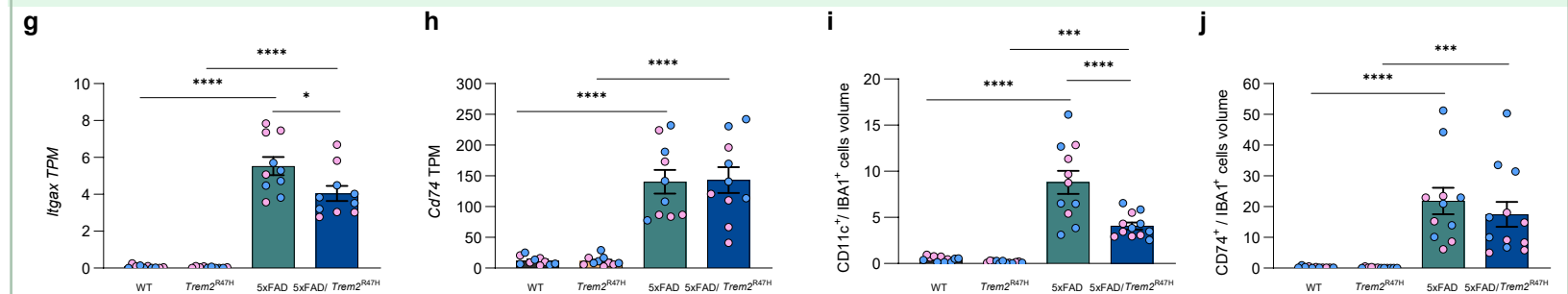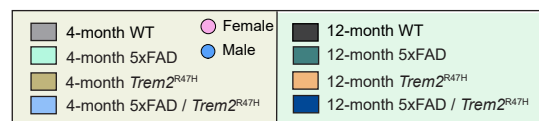

Supplement: Supplementary file 15 — Additional file 15: Supplemental Figure 14. Trem2R47H alters expression of Itgax and Cd74 in an age-dependent mannera, b Representative confocal images of hippocampal subiculum in wild-type, Trem2R47H, 5xFAD, and 5xFAD/Trem2R47H mice at (a) 4- and (b) 12-months old, stained with Amylo-Glo for dense-core plaques (green), immunolabeled with IBA1 for microglia (blue), CD11c (white), and CD74 (red) confirms reduced expression of Itgax (CD11c) and CD74. c, d 4-month TPM values of Itgax and Cd74 plotted as bar graphs showed decreased expression between 5xFAD and 5xFAD/Trem2R47H in both (c) Itgax and (d) Cd74. e, f Quantification of colocalization of IBA1 and (e) CD11c and (f) CD74 normalized to total IBA1 volume confirmed reduction in the respective gene expression. g, h 12-month-old TPM values of Itgax and Cd74 from bulk cortical RNA sequencing data revealed a decrease in (g) Itgax but not (h) Cd74. i, j Quantification of 12-month colocalization of IBA1 and (i) CD11c and (j) CD74 normalized to total IBA1 volume confirmed changes in the respective gene expression. n=10-12. Data are represented as mean ± SEM. Two-way ANOVA followed by Tukey’s post hoc tests to examine biologically relevant interactions. Statistical significance is denoted by *p<0.05, **p<0.01, ***p<0.001, ****p<0.0001. [file 13024_2023_598_MOESM15_ESM.pdf]
