# Supplementary material for: A Trem2R47H mouse model without cryptic splicing drives age- and disease-dependent tissue damage and synaptic loss in response to plaques
Source: Mol Neurodegener. 2023 Feb 17;18:12. doi: 10.1186/s13024-023-00598-4 (PMC9938579; doi:10.1186/s13024-023-00598-4)
Supplement: Supplementary file 16 — Additional file 16: Supplemental Figure 15. CRISPR-generated Trem2R47H alleles and their impact on induction of cryptic splicing events. DNA sequence alignment of part of exon 2 of mouse (Mm) Trem2 from wild-type and four CRISPR-generated R47H encoding alleles (from top to bottom, Cheng-Hathaway et. al [34], Trem2R47H CSS(JAX 027918), Xiang et. al [35] and Trem2R47H NSS (JAX 034036, this study). The R47 coding triplet and amino acid in mouse is shown in green, with the G to A transition that changes the R to H in each CRISPR allele shown in red. Blue nucleotides in each CRISPR-generated allele denote synonymous DNA bases co-introduced with the G to A transition. The equivalent region of human (Hs) TREM2 is shown at the top with nucleotide differences between mouse and human in brown. A summary of the presence of cryptic splicing and quantity of transcripts relative to the wild type allele is shown at the right of each CRISPR-generated allele (data from this study and [34, 35]. Evidence for cryptic splicing from the R47H Trem2 allele described in Cheng-Hathaway et. al [34] is from transfection of cells with recombinant DNA expression constructs as reported [35]. The underlined AG dinucleotide denotes the end of the “intronic” sequence removed in events using the cryptic splice site (identified in Xiang et. al [35] and this study) with the corresponding underlined TGA stop codon produced by the cryptic splice event. The bottom of the figure shows a consensus sequence for a splice branch point (YUVAY; Y = T or C; V = A, C or G [73]) and a polypyrimidine tract, with bases matching the wildtype mouse Trem2 sequence in green, which may stimulate cryptic splicing. Splice branch sequences in mammals are highly degenerate [74, 75]. Comparison of the sequence of each CRISPR-generated mouse R47H allele combined with results from studies using a cell culture-based assay, including analysis of the related human DNA sequence [35], does not provide clear insight into why transcrip [file 13024_2023_598_MOESM16_ESM.pdf]

Sup. Fig. 15

		splicing	expression
Hs	TCCCTGCAGGTGTCTTGCCCCTATGACTCCATGAAGCACTGGGGAGGCGCAAGGCCTGGTGCCGCCAGCTGGGAGAGAAG		
Mm AA	-S--L--R--V--S--C--T--Y--D--A--L--K--H--W--G--R--R--K--A--W--C--R--Q--L--G--E--E-		
Mm wild type	TCCTTGAGGGTGTCATGTACTTATGACGCCTTGAAGCACTGGGGAGACGCAAGGCCTGGTGTCGGCAGCTGGGTGAGGAG		
Cheng-Hathaway *et. al*	TCCTTGAGGGTGTCATGTACTTATGACGCCTTGAAGCACTGGGGAGACACAAGGCTTGGTGTCGGCAGCTGGGTGAGGAG	cryptic	reduced
JAX 027918 R47H CSS	TCCTTGAGGGTGTCATGTACTTATGACGCCTTGAAGCACTGGGGAGACACAAAGCATGGTGTCGGCAGCTGGGTGAGGAG	cryptic	reduced
Xiang *et. al*	TCCTTGAGGGTGTCATGTACTTATGACGCCTTGAAGCACTGGGTCGACACAAAGCCTGGTGTCGGCAGCTGGGTGAGGAG	cryptic	reduced
JAX 034036 R47H NSS	TCGTTAAGGGTATCCTGCACTTATGACGCGTTGAAACATTGGGGCAGACATAAGGCCTTGTGTCGGCAGCTGGGTGAGGAG	normal	normal

YUVAY YYYYYYYYYYNCAGGNN cryptic

intron | exon
