# Supplementary material for: A Trem2R47H mouse model without cryptic splicing drives age- and disease-dependent tissue damage and synaptic loss in response to plaques
Source: Mol Neurodegener. 2023 Feb 17;18:12. doi: 10.1186/s13024-023-00598-4 (PMC9938579; doi:10.1186/s13024-023-00598-4)
Supplement: Supplementary file 17 — Additional file 17: Supplemental Table 1. Primers for PCR amplification and sequencing for off-target analysis. Forward (For) and reverse (Rev) primer sequences are listed for each potential off-target site. The off-target code corresponds to the panels in Supplemental Fig.2. [file 13024_2023_598_MOESM17_ESM.pdf]

Supplemental Table 1: Primers for PCR amplification and sequencing for off-target analysis.

Off-target code	Primer Name	Primer Sequence (5' - 3')	Product size	Chromosome 17 locus
**a**	A For	ACCCACACTGGAGAGATACTG	382 bp	*Pacrg*, intron 4 (175kb intron)
	A Rev	CGTGGGAATGGGAACAGTAATG		
**b**	F For	CCCCTTCTGCTTGCTTCATAC	286 bp	Intergenic between *Cntnap5c* and *A330072L02Rik*
	F Rev	AGCTTGTTGCCCACTTGTAG		
**c**	B For	CCCTGTCTGAGCAGATGATTTAG	354 bp	*Zfp945*, Exon 1
	B Rev	CCTCTGACCTGGGAGGTTC		
**d**	G For	GGCAGGCTGGAAGGAAATAAG	704 bp	*Ndufv2*, Intron 2 (9kb intron)
	G Rev	GCAAACCAAGCCATCACTTTAC		
**e**	C For	AAACACCGGCCTCAGAAAG	365 bp	*Wiz*, Exon 7
	C Rev	AGCAGCACTCACTGTTGATATT		
**f**	H For	ATCCACTCTGTTGAGGGAAAG	274 bp	*Ptprm*, intron 15 (73kb intron)
	H Rev	AGTGGCCTTAGGAGGTAGAG		
**g**	D For	CAGACCCATCCTGTAGACTTG	1001 bp	Intergenic between *Mrpl14* and *Vegfa*
	D Rev	CTCAGGGTTACATGCCACTAC		
**h**	I For	GGGACCAAGGGAGTATTTCATAG	473 bp	Intergenic between *Lrrc30* and *Lama1*
	I Rev	TCTCCTTTCTCCAATCTCTCCTC		
**i**	E For	CCTTTCCTGGTGCTTCTCTTC	485 bp	*Tfeb*, intron 1 (45kb intron)
	E Rev	ACTCAAGCTGAAGGCCAAAG		
**J**	J For	GGGACCAAGGGAGTATTTCATAG	502 bp	*Clip4*, intron 1 (20kb intron)
	J Rev	GTGCCAGAGATGGTTCTACAG		
**K**	K For	ACTCCCTAAGAAAGAAGAACAGTAAG	549 bp	*Prkce*, intron 1 (180kb intron)
	K Rev	GTTCCTCAAGAGACACAAGGAG		
