# Supplementary material for: A Trem2R47H mouse model without cryptic splicing drives age- and disease-dependent tissue damage and synaptic loss in response to plaques
Source: Mol Neurodegener. 2023 Feb 17;18:12. doi: 10.1186/s13024-023-00598-4 (PMC9938579; doi:10.1186/s13024-023-00598-4)
Supplement: Supplementary file 11 — Additional file 11: Supplemental Figure 10. Trem2R47H affects dystrophic neurites and axonal damage in an age- and sex-specific manner. a-d Sex-separate quantification of LAMP1+ volume normalized to plaque volume at (a,b) 4 months and (c,d) 12 months. e-j Measurement of NfL in (e,f) plasma, (g,h) soluble and (i,j) insoluble brain fraction at 4 and 12 months separated by sex. k-n Quantification of (k,l) number and (m-n) total NfL+ spheroid volume in the subiculum at 4 and 12 months compared between genotypes for each sex. n=5-6. Data are represented as mean ± SEM. Student’s t-test. Two-way ANOVA followed by Tukey’s post hoc tests to examine biologically relevant interactions. Statistical significance is denoted by *p<0.05, **p<0.01, ***p<0.001, ****p<0.0001. [file 13024_2023_598_MOESM11_ESM.pdf]

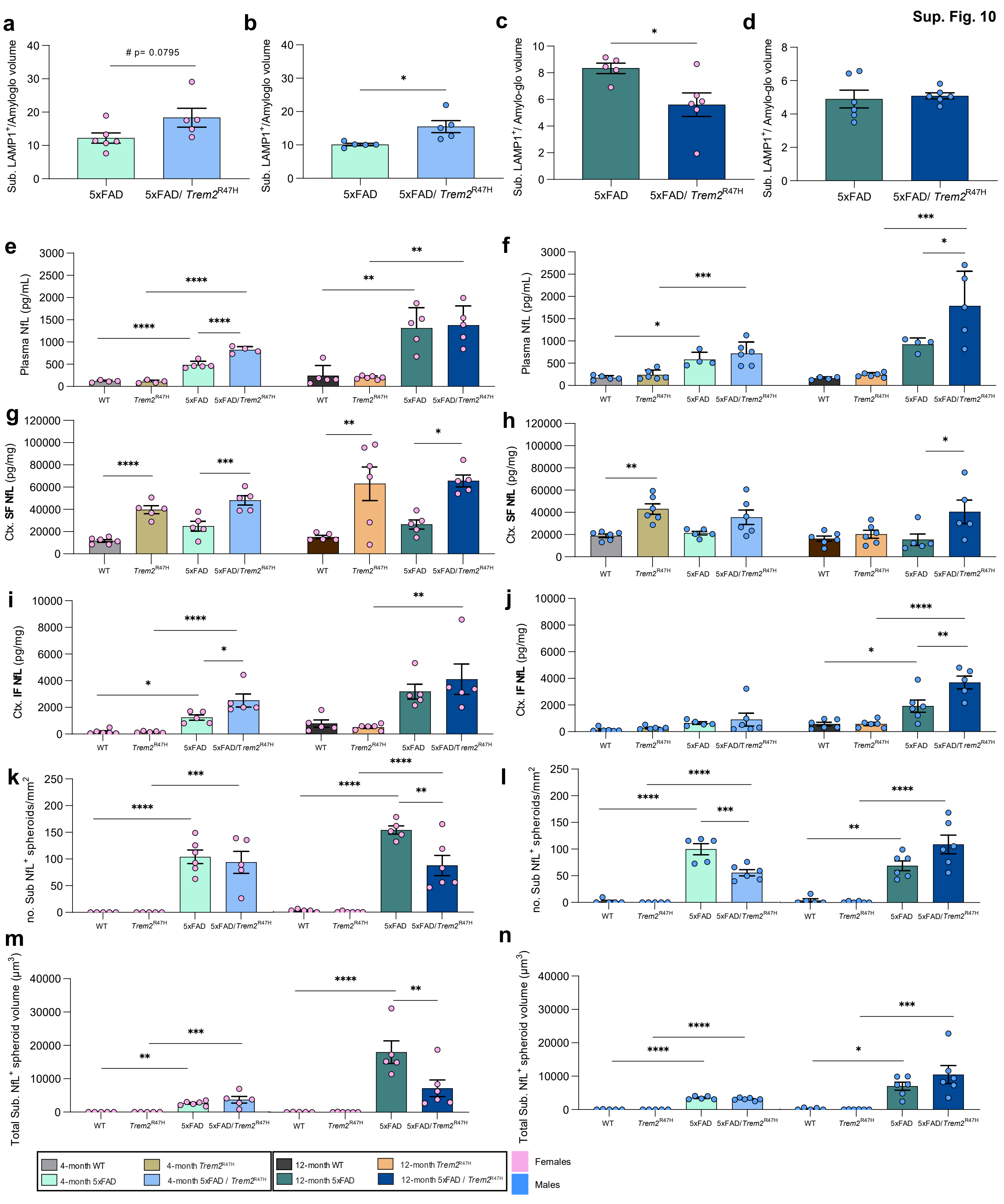
Sup. Fig. 10
a
b
c
d
Sub. LAMP1+/Amyloglo volume
Sub. LAMP1+/ Amylo-glo volume
# p= 0.0795
5xFAD
5xFAD/ Trem2R47H
e
f
Plasma NfL (pg/mL)
g
h
Ctx. SF NfL (pg/mg)
i
j
Ctx. IF NfL (pg/mg)
k
l
no. Sub NfL+ spheroids/mm2
m
n
Total Sub. NfL+ spheroid volume (µm3)
WT
Trem2R47H
5xFAD
5xFAD/Trem2R47H
4-month WT
4-month Trem2R47H
4-month 5xFAD
4-month 5xFAD / Trem2R47H
12-month WT
12-month Trem2R47H
12-month 5xFAD
12-month 5xFAD / Trem2R47H
Females
Males
