# Supplementary material for: A Trem2R47H mouse model without cryptic splicing drives age- and disease-dependent tissue damage and synaptic loss in response to plaques
Source: Mol Neurodegener. 2023 Feb 17;18:12. doi: 10.1186/s13024-023-00598-4 (PMC9938579; doi:10.1186/s13024-023-00598-4)
Supplement: Supplementary file 18 — Additional file 18: Supplemental Table 2. Location of potential off-target sites for crRNA TMF1342 on mouse chromosome 17. The sequence of each of the 11 potential off-target sites on mouse chromosome 17 is listed (GRCm38/mm10 nucleotide numbering), as well as the intended target site within Trem2. Mismatches to the crRNA guide are indicated by lowercase bold letters. Blue text denotes the 8-nucleotide seed region proximal to the PAM site. The vertical line at the 3’ end of each target or off-target site indicates the boundary between the gRNA target and the adjacent Cas9 PAM (NGG) sequence. The column labeled NSS vs WT p value lists the p-value for gene expression difference for each of the 11 potential off-target genes in whole brain between 15-week-old mice (n=8 for WT and n=4 for Trem2R47H NSS, all males, analyzed via bulk RNA-seq (edgeR, p-values stated). [file 13024_2023_598_MOESM18_ESM.pdf]

Supplemental Table 2: Location of potential off-target sites for crRNA TMF1342 on mouse chromosome 17

Genomic DNA (11 base seed)	Position	Strand	Mis-matches	Nearest gene	Off-target code	NSS vs WT p value
acAGCcCTGGGGGAGAgGCA|AGG	10,545,906	+	4	*Pacrg*, non-conserved region within intron 4 (175kb intron)	a	0.264
GAAGCAgTGtGGGAcACtCA|GGG	58,666,935	-	4	Intergenic between *Cntnap5c* and *A330072L02Rik*	b	0.46
GAAGaACTcaGGGAGcCGCA|CGG	22,867,101	-	4	*Zfp945*, Exon 1	c	0.743
GAAGaACTGGGatAGAaGCA|GGG	66,097,472	-	4	*Ndufv2*, Intron 2 (9kb intron)	d	0.663
GAAGtgCTGGGGGAGAacCA|GGG	32,356,988	-	4	*Wiz*, Exon 7	e	0.635
tAAGCACTGGcaGAGAaGCA|GGG	66,865,733	+	4	*Ptprm*, intron 15 (73kb intron)	f	0.509
GAAGCACTtGGGGAGtCaCA|AGG	45,861,722	-	3	Intergenic between *Mrpl14* and *Vegfa*	g	0.376, 0.816
GAAGCAgTGtGGaAGAtGCA|GGG	67,693,239	-	4	Intergenic between *Lrrc30* and *Lama1*	h	0.37
GcAGCACgGGGGGAGACtCc|GGG	47,764,151	-	4	*Tfeb*, intron 1 (45kb intron)	i	0.694
ccAGCACTGGGGcAGACaCA|GGG	71,776,150	-	4	*Clip4*, intron 1 (20kb intron)	j	0.712
GAAaCACTtGGGGgGAgGCA|GGG	86,300,554	+	4	*Prkce*, intron 1 (180kb intron)	k	0.486
GAAGCACTGGGGGAGACGCA|AGG	48,348,538	+	0	*Trem2*, Exon 2		
